# A rigorous framework for an improved Messinger/Myers model of ice accretion under conditions of variable property and unsteady aircraft icing

**DOI:** 10.1098/rsta.2024.0367

**Published:** 2025-07-17

**Authors:** Hashnayne Ahmed, Arash Shad, Nadim Zgheib, S. A. Sherif, S. Balachandar

**Affiliations:** ^1^ Department of Mechanical and Aerospace Engineering, University of Florida, Gainesville, FL 32611, USA; ^2^ Department of Mechanical Engineering & Institute for Advanced Manufacturing, The University of Texas Rio Grande Valley, Edinburg, TX 78539, USA

**Keywords:** Messinger model, Myers model, ice accretion, aircraft icing, atmospheric icing, supercooled droplets, rime ice, glaze ice, unsteady, variable properties

## Abstract

We analyse the Messinger/Myers model by critically evaluating simplifying assumptions through a rigorous formulation of the rime ice accretion process. We explore the effects of both constant and variable ice density and thermal conductivity, along with the effects of sublimation from the ice surface. The effects of key factors such as droplet impact rate, ambient temperature relative to the freezing temperature and the temperature difference between the ambient air and the airfoil surface are examined. Under these varying conditions, the present rigorous formulation is used to assess the significance of unsteady effects, variable ice properties and sublimation. We observe that the Myers model performs remarkably well in certain icing situations and analyse the reasons for this strong performance. We also show that partially relaxing the model’s assumptions can lead to poorer performance. The Myers model can lead to overprediction of ice surface temperature and correspondingly underprediction of transition time under conditions of relatively weak sublimation and surface cooling. A modified Myers model is presented, which can be used to recover near-perfect results under widely varying icing conditions of relevance.

This article is part of the theme issue ‘Heat and mass transfer in frost and ice’.

## 1. Introduction

Aircraft icing poses significant challenges to aviation safety and efficiency, particularly in regions characterized by cold weather conditions. The formation of ice on aircraft surfaces alters aerodynamic performance, leading to reduced lift, increased drag and potential loss of control. Accurate prediction and mitigation of icing phenomena are therefore crucial for ensuring safe flight operations. Traditional approaches to aircraft icing prediction in the past often relied on empirical approaches that lack the ability to capture the complex dynamics of ice formation and accretion. To address this limitation, advanced computational techniques, such as multiphase simulation, have emerged as promising tools for enhancing our understanding of icing processes [1].

Aircraft icing can be categorized into different types, each with unique characteristics and hazards [2,3]. Rime ice generally forms when the ambient temperature falls in the range of −15°C to −20°C. At such low temperatures, supercooled water droplets freeze upon impact, creating a rough, milky and opaque coating. This ice coating increases drag and reduces lift, thereby significantly impairing aircraft performance and control. Glaze ice, also known as clear ice, forms at ambient temperatures between 2°C and −10°C when larger droplets spread out before freezing, resulting in a smooth, transparent layer. This layer drastically alters aerodynamic properties, often forming complex shapes like horns that significantly disrupt airflow, making detection and removal more challenging. Mixed ice occurs between −10°C and −15°C, combining characteristics of both rime and glaze ice. It forms rapidly when ice particles become embedded in clear ice, resulting in a hard, rough conglomerate that presents unique challenges for detection and removal due to its heterogeneous structure. The effects of icing include reduced lift, increased drag, increased weight and increased thrust requirements, all of which can severely impact aircraft performance and safety. A deeper understanding of the various types of ice (rime versus glaze) and the ability to accurately predict their formation are crucial for developing effective de-icing strategies and ensuring flight safety.

Traditional empirical approaches are limited by their inability to dynamically adjust to conditions that vary both in time and across the icing surface of the airfoil, underscoring the need for more advanced approaches that provide accurate, real-time predictions [4]. The development of multiphase simulations marked a significant improvement in the field of aircraft icing prediction by considering the simultaneous presence and interaction of multiple phases, providing a more detailed and realistic representation of icing processes. Numerical simulations have further enhanced our capabilities by offering high-resolution depictions of fluid dynamics around aircraft surfaces, allowing for precise predictions of ice formation patterns [5]. Multiphase flow simulations enable accurate representation of complex flow fields and phase changes at the microscale, capturing the intricate interactions between water droplets and ice particles as they form and accumulate on aircraft surfaces [6]. Lagrangian droplet tracking has emerged as a powerful technique within these advanced models, offering detailed simulations of individual droplet trajectories and their interactions with the aircraft surface [7,8]. The Lagrangian approach is able to better capture the effect of turbulence and the stochastic nature of droplet impact and freeze behaviour, which traditional Eulerian methods have to model [9]. The ability to track individual droplets makes the Lagrangian approach particularly useful for understanding the microphysical processes involved in ice accretion, leading to more accurate predictions.

Modern computational approaches to airfoil icing prediction consist of two interconnected steps. First, the flow around the airfoil is simulated along with the trajectories of supercooled water droplets to determine the rate at which droplets impinge on different portions of the airfoil. Second, this droplet impingement rate is used to perform a local mass and heat transfer balance on the airfoil surface, assessing both the nature (rime or glaze) and the quantity of ice accretion. These two processes can be referred to as the aerodynamic *flow and impingement model* and the thermodynamic *icing model*. The thermodynamic icing model is typically one-dimensional and applied locally at every point on the airfoil surface with airflow and droplet impingement input from the three-dimensional and time-dependent flow simulation. The two models are coupled since the accumulated ice alters the aerodynamic flow around the airfoil and the subsequent droplet impingement rate, which in turn affects the rate of ice accretion and further modifies the airfoil surface.

This paper focuses on analysing icing models and critically evaluating their common assumptions through a rigorous formulation of the rime ice accumulation process. One of the earliest and most influential icing models is the Messinger model, developed in 1953, which is based on a global heat and mass balance of a layer of ice [10]. Some of the assumptions of the original Messinger model have been relaxed by incorporating more detailed aerodynamic and thermodynamic considerations, thereby allowing for a smoother transition between rime and glaze ice [11–14]. Here we will refer to the extended model discussed by Myers [11] as the Myers model.

There has been continued research on the Messinger/Myers model. For example, the PolimICE model explicitly accounted for sublimation and introduced a parabolic temperature profile to simulate the mass and heat transfer dynamics between rime and glaze ice accretion [15]. As a further refinement, the ice accretion solution was enhanced within PolimICE by considering the unsteady Stefan problem [16]. There have also been several other recent developments in the glaze icing model with the presence of a thin water layer on top of the ice layer [17–21].

In general, the following three common assumptions are made in the formulation of icing models: (i) steady heat conduction is often assumed within the ice layer, with the additional assumption of constant thermal conductivity. This results in a linear temperature variation across the ice layer. Unsteadiness arises because the ice layer thickens and the ice surface temperature varies over time. An analysis of unsteady heat conduction within the ice layer using the self-similarity assumption [16] indicated that the effect of unsteadiness is generally small. (ii) Ice properties, such as ice density and thermal conductivity, vary across the thickness of the ice layer, since as ice accretion proceeds, the surface temperature where new ice forms increases steadily from the initial airfoil temperature up to the melting point. As a result, ice density and thermal conductivity increase from the airfoil surface towards the ice surface. However, many ice models ignore this depth-dependent variation in the ice properties. By assuming linear variations in ice properties, Zhang *et al*. [22] obtained more complex temperature profiles within the ice layer. (iii) Other common assumptions often used in the icing model include ignoring the effect of ice sublimation and the simplified treatment of aerodynamic heating.

Here, we will present a rigorous formulation of the icing process that is appropriate for rime ice without the presence of a water layer. The analysis includes the unsteady effects arising from both ice growth and ice surface temperature variation. The solution obtained with constant ice properties helps evaluate the importance of unsteadiness. We then use asymptotic analysis to obtain a rigorous solution that includes variable ice properties and also the effect of sublimation. The two analyses are then combined to obtain a solution that includes the effects of unsteadiness, variable properties and sublimation from the ice surface.

The external conditions of airfoil icing vary significantly based on several factors, including the rate of droplet mass impact and ice accretion (ranging from very slow to very rapid), droplet impact velocity, the ambient and airfoil surface temperatures relative to freezing and free-stream airflow velocity. Under these varying conditions, we use the present icing formulation to investigate the importance of incorporating unsteady effects, variable properties and sublimation in the icing model. Three non-dimensional parameters characterizing the importance of variable ice density, variable thermal conductivity and sublimation are defined, and their range of possible values is established. The following three regimes of rime ice accretion are identified: (R1) rime ice accumulates until it reaches a steady state, where its thickness and temperature profile no longer vary, with the latter staying below the melting point; (R2) rime ice continues to accumulate at a constant rate with the ice surface temperature reaching a steady value below the melting point; (R3) rime ice transitions to glaze ice after a finite time period. The conditions under which these three regimes are observed are also established.

Our observations indicate that the Myers icing model [11], which is an extension of the original Messinger model, performs reasonably well in predicting the time evolution of the ice thickness and ice surface temperature. In fact, surprisingly, the Myers model outperforms other formulations that attempt to include additional physics in a partial manner. We analyse the reasons for this good performance. However, the Myers model overestimates the increase in ice surface temperature, leading to an underestimation of the critical transition time to glaze ice by approximately 20% under certain conditions. Using the short- and long-time asymptotic analysis, we propose a modified Myers (MM) model that is quite accurate in predicting ice thickness and ice surface temperature over a wide range of conditions, taking into account variable ice properties and the effect of sublimation. (The nomenclature associated with the contents of this paper is given in table 1.)

**Table 1.** Nomenclature

${\dot{h}}_{im}$	ice thickness increase rate, ${\text{m s}}^{-1}$
${\dot{m}}_{s}$	mass sublimation rate, ${\text{kg m}}^{-2}{\text{s}}^{-1}$
L	characteristic length scale, m
T	characteristic time scale, s
$\tilde{z}$	non-dimensional thickness coordinate
A	non-dimensional heat loss coefficient
a	speed of sound, m s^−1^
$A^{\prime }$	total heat loss coefficient, W m^−2^ K^−1^
B	non-dimensional heat addition coefficient
$B^{\prime }$	total heat addition, W m^−2^
$C_{p}$	specific heat capacity, J kg^−1^ K^−1^
$e_{0}$	vapour pressure constant, Pa K^−1^
h	ice thickness, m
k	thermal conductivity, W m^−1^ K^−1^
$k_{i0}$	initial thermal conductivity of ice, W m^−1^ K^−1^
$L_{f}$	latent heat of fusion, J kg^−1^
$L_{s}$	latent heat of sublimation, J kg^−1^
Le	Lewis number
$p_{0}$	ambient pressure, Pa
$Q_{a}$	aerodynamic heat, W m^−2^
$Q_{c}$	convective heat, W m^−2^
$q_{c}$	convective heat coefficient, W m^−2^ K^−1^
$Q_{d}$	sensible heat, W m^−2^
$q_{d}$	sensible heat coefficient, W m^−2^ K^−1^
$Q_{k}$	kinetic heat, W m^−2^
$Q_{L}$	latent heat, W m^−2^
$Q_{r}$	radiative heat loss, W m^−2^
$q_{r}$	radiative heat coefficient, W m^−2^ K^−1^
$Q_{s}$	sublimation heat loss, W m^−2^
$q_{s}$	sublimation heat coefficient, W m^−2^ K^−1^
r	adiabatic recovery factor
T	temperature, K
t	time, s
$t_{c}$	critical transition time, s
$U_{\infty }$	free-stream velocity, m s^−1^
$v_{im}$	droplet impact velocity, m s^−1^
z	thickness coordinate, m
${\dot{m}}_{im}$	mass impingement rate, kg m^−2^ s^−1^
$\hat{h}$	non-dimensional ice thickness
Greek symbols	
α	weighting coefficient
$\chi_{s}$	mass sublimation coefficient, kg m^−2^ s^−1^ K^−1^
$\Delta_{\rho }$	density variation, kg m^−3^
$\Delta_{k}$	conductivity variation, W m^−1^ K^−1^
γ	specific heat ratio of air
ρ	density, kg m^−3^
$\rho_{i0}$	initial density of ice, kg m^−3^
σ	Stefan–Boltzmann constant, W m^−2^K^−4^
τ	non-dimensional time
θ	non-dimensional temperature
$\theta^{*}$	non-dimensional temperature at formation
ε	emissivity
${\hat{\chi }}_{s}$	non-dimensional sublimation coefficient
${\hat{\Delta }}_{\rho }$	normalized density variation
${\hat{\Delta }}_{k}$	normalized conductivity variation
ζ	non-dimensional thickness coordinate
subscripts	
0	initial
a	ambient
c	critical
ef	effective
f	freezing
g	glaze
I	interface
i	ice
im	impingement
M	Myers
MM	modified Myers
P	power series
s	surface
stg	stagnation
tr	transition
w	water

## 2. Rime ice formulation—constant ice properties

**Figure 1.**
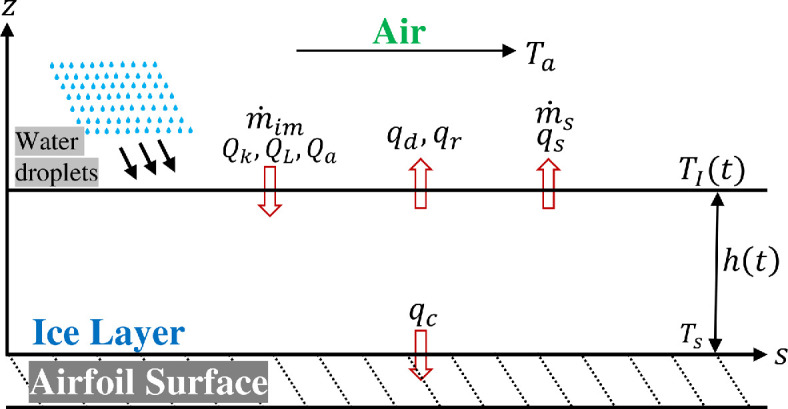
Schematic of rime ice growth within a control volume at a local point on an airfoil surface showing key variables and associated heat and mass transfer terms. Fluxes denoted by Q represent direct energy contributions, while terms with *q* are heat transfer coefficients used in the model formulation.


In this section, we aim to develop a rigorous formulation of rime ice growth, i.e. in the absence of a liquid water layer on top of the ice surface. The absence of the liquid layer on top of the ice layer simplifies the problem. Nevertheless, we want to start the rigorous formulation in this simpler limit. As shown in figure 1, the airfoil surface is maintained at a constant temperature $T_{s}$, while the far-field air in the ambient is maintained at a constant temperature $T_{a}$, which can be taken to be the same as the airfoil surface temperature. The time-dependent rime ice thickness and surface temperature are denoted as h(t) and $T_{I}(t)$, where the subscript ‘I’ denotes the ‘ice–air interface’. Ignoring any lateral variation, we consider the unsteady one-dimensional thermal conduction problem. The evolution of the ice temperature T(z,t) is then governed by

(2.1) $$\rho_{i}C_{p,i}\frac{\partial T}{\partial t}=\frac{\partial }{\partial z}\left(k_{i}\frac{\partial T}{\partial z}\right)\;\text{ with }\;0\leq z\leq h(t),$$

where $\rho_{i}$, $k_{i}$ and $C_{p,i}$ are the density, thermal conductivity and specific heat capacity of ice, which in this section are taken to be depth-independent constants. The boundary conditions at the airfoil surface (z=0) and at the ice–air interface (z=h(t)) are governed by


(2.2) $$T(z=0,t)=T_{s}\;\text{ and }\;-{k_{i}\frac{\partial T}{\partial z}|}_{z=h(t)}=\underset{=A^{\prime }}{\underbrace{\left(q_{c}+q_{d}\right)}}\left(T_{I}-T_{a}\right)-\underset{=B^{\prime }}{\underbrace{\left(Q_{k}+Q_{L}+Q_{a}\right)}}.$$

The second boundary condition at the ice–air interface is the Stefan equation of thermal balance, where the first term on the right-hand side corresponds to different heat removal processes that are proportional to the temperature difference $\left(T_{I}-T_{a}\right)$ between the ice surface and the ambient. In this term, $q_{c}$ and $q_{d}$ represent the convective heat transfer from the ice layer to the ambient and the sensible heat needed to warm the impinging droplets, respectively. Both $q_{c}$ and $q_{d}$ measure heat loss for a unit temperature difference between the ice surface and the ambient. The two heat loss coefficients together will be denoted as $A^{\prime }$. The second term on the right-hand side corresponds to heat addition through different mechanisms: kinetic energy of the impinging droplets ($Q_{k}$), latent heat released during the freezing of the droplets ($Q_{L}$) and aerodynamic heating ($Q_{a}$). Together these terms are denoted by $B^{\prime }$. The explicit expressions for all these terms are well-established and discussed in the literature.

In formulating equation (2.2), we have ignored the heat loss effects of sublimation and radiation, primarily to keep the analysis of this section simple. Both these effects can be included, and the analysis will be redone in the following section, where we will also relax the assumption of constant properties. Heat loss due to sublimation is proportional to the temperature difference between the ice surface and the ambient. It can therefore be easily added to the first term on the right-hand side of equation (2.2) with the inclusion of $q_{s}(T_{I}-T_{a})$. However, sublimation complicates the analysis by altering the mass balance. Radiative loss depends on the fourth power of the ice surface temperature and the ambient temperature, with the temperature difference between the ice surface and the ambient being typically small. As a result, radiative heat loss can be approximately expressed as $q_{r}(T_{I}-Ta)$ (see appendix A for details). The contribution of radiation to heat balance is, however, usually quite small. In summary, with the inclusion of sublimation and radiation, the first term on the right-hand side of equation (2.2) would be expressed as $A^{\prime }=q_{c}+q_{d}+q_{s}+q_{r}$.

To accurately account for the time-varying thickness of the ice layer, we introduce a non-dimensional vertical coordinate ζ=z/h(t), where it should be noted that a fixed z location in the ice layer will now correspond to time-varying ζ that depends on the ice thickness. The important consequences of this change of variable are the transformations (∂T/∂t)→(∂T/∂t)−(ζ/t)(∂T/∂ζ) and (∂T/∂z)→(1/h(t))(∂T/∂ζ). Substituting, we obtain the modified unsteady equation as


(2.3) $$\rho_{i}C_{p,i}\left(\frac{\partial T}{\partial t}-\frac{\zeta }{t}\frac{\partial T}{\partial \zeta }\right)=\frac{k_{i}}{h^{2}(t)}\frac{\partial^{2}T}{\partial \zeta^{2}}\;\text{ with }\;0\leq \zeta \leq 1,$$

where we have taken the thermal conductivity of ice to be a constant.

The droplet mass impingement rate per unit area of the airfoil surface per unit time, ${\dot{m}}_{im}$, is an important input to the icing model. We assume ${\dot{m}}_{im}$ to be a constant over time, and in the absence of sublimation, the ice layer thickness grows linearly. The mass balance equation of ice accretion becomes


(2.4) $$\rho_{i}\frac{\text{d}h}{\text{d}t}={\dot{m}}_{im}\;\Rightarrow \;h(t)=\underset{={\dot{h}}_{im}}{\underbrace{\left({\dot{m}}_{im}/\rho_{i}\right)}}\;t\;.$$

Written this way, ${\dot{h}}_{im}$ represents the rate at which the ice thickness increases. This allows the introduction of $L=k_{i}/(C_{p,i}{\dot{m}}_{im})$ and $T=k_{i}\rho_{i}/(C_{p,i}{\dot{m}}_{im}^{2})$ as the length and time scales. For example, an ice layer growth rate of 0.02 mm s^−1^ at an ice layer temperature of about −10°C yields length and time scales of L=57 mm and T=2866 s. The non-dimensional time, τ, and the non-dimensional temperature, θ, are now defined as


(2.5) $$\tau =\frac{t}{T}\;\text{ and }\;\theta =\frac{T-T_{s}}{T_{f}-T_{s}},$$

where $T_{f}=273.15$ K is the melting temperature of ice. Non-dimensionalizing, we obtain the mass balance equation as


(2.6) $$\begin{array}{lcr} & \hat{h}(\tau )=\frac{h}{L}=\tau \;. & \end{array}$$

Substituting equations (2.6) and (2.5) into the unsteady heat conduction equation and boundary conditions, we obtain


(2.7) $$\tau^{2}\frac{\partial \theta }{\partial \tau }-\zeta \tau \frac{\partial \theta }{\partial \zeta }-\frac{\partial^{2}\theta }{\partial \zeta^{2}}=0,\;\theta (\zeta =0,\tau )=0,\;\text{ and }{\;\frac{\partial \theta }{\partial \zeta }|}_{\zeta =1}=\tau \left(B-{A\theta |}_{\zeta =1}\right),$$

where


(2.8) $$A=\frac{A^{\prime }}{{\dot{m}}_{im}C_{p,i}}\;\text{ and }\;B=\frac{B^{\prime }}{{\dot{m}}_{im}C_{p,i}\left(T_{f}-T_{s}\right)}+\frac{A^{\prime }}{{\dot{m}}_{im}C_{p,i}}\underset{=\theta_{a}}{\underbrace{\left(\frac{T_{a}-T_{s}}{T_{f}-T_{s}}\right)}}.$$

### (a) Interpretation and estimation of the coefficients *A* and *B*

The non-dimensional coefficient A measures the rate of total heat loss from the ice surface due to convective cooling and droplet heating, relative to the sensible heat contained in the impinging droplets. Later in §3, we will also include the cooling effects due to sublimation and radiation to define $A=\left(q_{c}+q_{d}+q_{s}+q_{r})/({\dot{m}}_{im}C_{p,i}\right)$. The definition of these various terms is presented in appendix A. From the definition, it can be seen that $q_{d}=1$ and thus the minimum value of A is unity. In the definition of B, the effect of the second term is generally small since we can expect $T_{a}\approx T_{s}$, and as a result, $\theta_{a}\approx 0$. From appendix A, it can be seen that the definition of B must also include the additional contribution of ${\dot{m}}_{im}(C_{p,w}-C_{p,i})(T_{a}-T_{f})$. Therefore, the non-dimensional coefficient B measures the rate of total heat addition relative to sensible heat contained in the impinging droplets, where the mechanisms of heat addition are kinetic heating, latent heat release due to freezing of the droplets, aerodynamic heating and residual sensible cooling due to droplets. It should be noted that an important contribution to $B^{\prime }$ comes from latent heat release from the freezing droplets. If we make the approximation $B\approx L_{f}/(C_{p,i}(T_{f}-T_{s}))$, then B falls within a range of 5.3–31.7 as the airfoil temperature varies from −30°C to −5°C.

**Figure 2.**
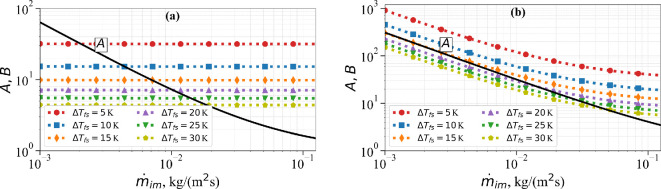
A (solid black line) and B (coloured dotted lines) versus mass impingement rate ${\dot{m}}_{im}$ for (a) case 1 and (b) case 2 for different $\Delta T_{fs}$.

From its definition, it can be seen that the value of A can vary over a wide range. A is small when the mass impingement rate of the droplets is large and vice versa. We now consider the following two different cases: (i) case 1: a low free-stream velocity of $U_{\infty }=10$ m s^−1^, low impingement velocity of $v_{im}=5$ m s^−1^ and low heat transfer coefficient of 70 W (m^−2^K^−1^), and (ii) case 2: a high free-stream velocity of $U_{\infty }=250$ m s^−1^, high impingement velocity of $v_{im}=100$ m s^−1^ and low heat transfer coefficient of 345 W (m^−2^K^−1^). Figure 2a shows a plot of A versus ${\dot{m}}_{im}$ for the low-speed case 1. The heat addition coefficient B also depends on the value of $\Delta T_{fs}=\left(T_{f}-T_{s}\right)$. So, figure 2a also shows plots of B versus ${\dot{m}}_{im}$ for six different values of $\Delta T_{fs}=5$, 10, 15, 20, 25 and 30 K. At the highest mass impingement rate presently considered, which is in excess of ${\dot{m}}_{im}=0.1$ kg (m^−2^s^−1^), A attains its smallest value of about 1.0. On the other hand, A attains its largest value for the smallest presently considered value of ${\dot{m}}_{im}=10^{-3}$ kg (m^−2^s^−1^), whereby A≈45. In the low-velocity case 1, B is nearly independent of ${\dot{m}}_{im}$ and increases from about 4 to 32 as the value of $\Delta T_{fs}$ increases. For any given temperature difference $\Delta T_{fs}$, to the left of the A versus ${\dot{m}}_{im}$ curve, i.e. for sufficiently small values of droplet mass impingement rate, the cooling effect will exceed the heating effect. On the other hand, a sufficiently strong droplet mass impingement rate will lead to B>A, and the heating effect will exceed the cooling effect. As we will see below, the transition and B<A and B>A impact the nature of long-time ice accretion.

The results for the high-velocity case 2 are shown in figure 2b. At the highest mass impingement rate considered, A reaches its smallest value of 4.57. On the other hand, with a decreasing mass impingement rate, A increases to about 457 for ${\dot{m}}_{im}=10^{-3}$ kg (m^−2^s^−1^). For large ${\dot{m}}_{im}$, the value of B is similar to its value in case 1. However, with decreasing droplet mass impingement rates, B increases to take values of about 200 and 1300 for $T_{f}-T_{s}=5$ and 30 K, respectively. Again, we can demarcate conditions under which surface cooling will dominate surface heating (i.e. B<A) and vice versa. In case 2, for the range of ${\dot{m}}_{im}$ considered, A never exceeds B for $\Delta T_{fs}=5$ and 10 K. In general, for sufficiently small droplet impingement rates and small values of $\Delta T_{fs}$, the cooling effect exceeds the heating effect.

### (b) Analytical solution

We now proceed to solve the governing equation and boundary conditions given in equation (2.7). An exact analytical expression that applies to all values of τ has not been obtained. Instead, we will obtain a numerical solution that is valid for all τ. Before proceeding to the numerical solution, we present short- and long-time analytical results.

In the short limit of t≪T (or τ≪1), we can seek an asymptotic power series solution of the form


(2.9) $$\begin{array}{lcr} & \theta (\zeta ,\tau )=\theta_{0}(\zeta )+\tau \theta_{1}(\zeta )+\tau^{2}\theta_{2}(\zeta )+\tau^{3}\theta_{3}(\zeta )+O(\tau^{4})\;. & \end{array}$$

Substituting the above-given expansion into the governing equation and the boundary conditions, solutions can be obtained for $\theta_{1}$, $\theta_{2}$, etc. Since we estimated T to be of the order of an hour, the asymptotic solution is expected to be accurate for the early time of the ice accretion process of the order of a few minutes. The asymptotic solution will be inaccurate beyond this early time limit. Similarly, the long-time steady-state solution can be obtained by ignoring the ∂θ/∂τ term in equation (2.7). These limiting solutions are given below


(2.10) $$\theta (\zeta ,\tau )=\left\{ \begin{array}{ll} \theta_{P\ll }(\zeta ,\tau )=B\zeta \tau -AB\zeta \tau^{2}+\left(\left(A^{2}B+\frac{AB}{2}\right)\zeta -\frac{AB}{6}\zeta^{3}\right)\tau^{3}+O\left(\tau^{4}\right), & \text{ if }\tau \ll 1\\ \theta_{P\gg }(\zeta ,\tau )=\frac{\tau B\mathrm{erf}(\sqrt{\tau /2}\zeta )}{\sqrt{2\tau /\pi }e^{-\tau /2}+A\tau \mathrm{erf}(\sqrt{\tau /2})}, & \text{ if }\tau \gg 1. \end{array}\right.$$

For future reference, we denote the two limiting analytical solutions as $\theta_{P\ll }(\zeta ,\tau )$ and $\theta_{P\gg }(\zeta ,\tau )$, where the letter ‘P’ in the subscript denotes that these are solutions of the partial differential equation (PDE) (2.7) without the assumption of a linear temperature profile. It should be noted that even though the time derivative has been ignored, $\theta_{P\gg }$ still depends on τ due to the dependence of the remaining terms and boundary conditions in equation (2.7) on τ. $\theta_{P\gg }$ can be Taylor series expanded in τ to obtain $\theta_{P\gg }∼B\zeta \tau -(A-(1/3))B\tau^{2}$. Comparing this with $\theta_{P\ll }$, we see that the PDE solution obtained by ignoring the time derivative deviates from the true small-time behaviour at $O(\tau^{2})$.

### (c) Messinger/Myers model

To evaluate the importance of rigorously including the effects of unsteadiness in the analysis, as was done in the above derivation, we must compare the results with those of the Myers model given in [11]. We will continue to ignore the effects of sublimation and radiative loss. In addition, the Myers model substantially simplified the problem by assuming the thermal profile within the ice layer to be linearly varying from $T_{s}$ at the airfoil surface to $T_{I}$ at the ice–air interface. From equation (2.10), it can be seen that except at very short times, there can be deviation from the linear temperature profile. Nevertheless, with the linear temperature profile, the temperature gradient on the left-hand side of the Stefan equation (equation (2.2)) can be approximated as $\left(T_{I}-T_{s})/({\dot{h}}_{im}t\right)$. Substituting into the Stefan equation and carrying out the non-dimensionalization, we can obtain the solution as written by Myers [11]. In current variables, the equations of non-dimensional ice thickness and ice surface temperature become


(2.11) $${\hat{h}}_{M}(\tau )=\tau \;\text{ and }\;\theta_{M}(\zeta ,\tau )=\frac{B{\hat{h}}_{M}\zeta }{1+A{\hat{h}}_{M}},$$

where the subscript ‘M’ is used to refer to the Myers solution.

**Figure 3.**
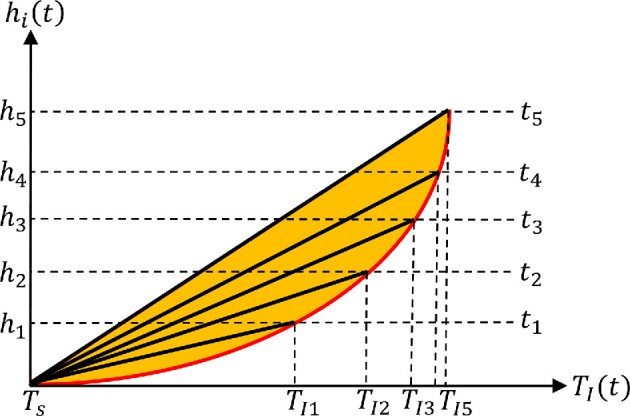
Temporal evolution of ice layer thickness $h_{i}(t)$ versus interface temperature $T_{I}(t)$ is shown in red. While the ice thickness grows steadily, the increase in surface temperature slows down due to increased heat loss. The black lines represent the linear temperature profiles within the ice layer at five different time instances, $t_{1},t_{2},t_{3},t_{4},t_{5}$. For example, a fresh layer of ice was deposited at time $t_{2}$ at $z=h_{2}$ and temperature $T_{I2}$. With the assumption of a linear temperature profile, as ice deposition proceeds, this layer of ice at $z=h_{2}$ decreases in temperature. The shaded area corresponds to sensible heat release.


The Stefan condition, since it is applied solely at the ice surface as the local energy balance, implicitly introduces another important approximation. Under constant ice accretion, the ice layer thickness grows linearly. In comparison, the increase in ice surface temperature is not linear, since the increase of $T_{I}$ slows down to approach $T_{f}$. Therefore, with the linear temperature profile within the ice layer, the thermal gradient $\left(T_{I}-T_{s})/({\dot{h}}_{im}t\right)$ decreases over time (figure 3). As a consequence, the ice temperature at any z is the highest when it initially forms, but decreases slowly as more ice deposits. In essence, as the ice layer grows, sensible heat is released, and its contribution is ignored when one considers only the Stefan condition. A careful reformulation that takes into account this sensible heat yields the following ODE for the ice surface temperature

(2.12) $$\frac{\tau }{2}\frac{\text{d}\theta_{O,I}}{\text{d}t}+\theta_{O,I}\left(\frac{1}{\tau }+(A-\frac{1}{2})\right)=B\;,$$

where the subscript ‘*_O_*, *_I_*’ represents the solution of the ordinary differential equation (ODE) for the ice–air interface temperature, from which the temperature within the ice layer can be obtained as $\theta_{O}(\zeta ,\tau )=\zeta \;\theta_{O,I}(\tau )$. As in the PDE, we have not obtained a general solution. The limiting solutions are obtained (i) for small τ as an asymptotic power series and (ii) for large τ when $\text{d}\theta_{O,I}/\text{d}t$ can be ignored. These two limiting solutions are expressed as


(2.13) $$\begin{array}{r} \theta_{O}(\zeta ,\tau )=\left\{ \begin{array}{ll} \theta_{O\ll }(\zeta ,\tau )=B\zeta \tau -AB\zeta \tau^{2}+O(\tau^{3}), & \text{if}\;\;\tau \ll 1\\ \theta_{O\gg }(\zeta ,\tau )=\frac{B\tau \zeta }{1+(A-\frac{1}{2})\tau }, & \text{if}\;\;\tau \gg 1\;. \end{array}\right. \end{array}$$

### (d) Comparison of the models

We first compare the different solutions in terms of their prediction of the ice surface temperature for the short-time asymptotic solution, which is valid for τ≪1, and for the long-time asymptotic solution in the limit of τ≫1. These results are presented in table 2. Several interesting conclusions emerge from the table. (i) First and foremost, the Myers solution, despite its assumption of a steady linear profile and ignoring the change in the sensible heat content of the previously deposited ice, is very accurate in predicting both the short-time behaviour and the long-time steady state. (ii) All the models correctly predict the leading order short-time solution to be Bτ. In other words, at the very early stages of ice growth, ice surface temperature increases linearly with time as $\text{d}\theta_{I}/\text{d}t=B$, and this behaviour is correctly captured by all the models. This indicates that the initial increase in ice surface temperature depends only on heat addition (B) and not on heat loss (A), which is due to our assumption of $T_{s}=T_{a}$. (iii) Ignoring the time derivative term in both the ODE and the PDE formulations leads to an incorrect evolution of the ice surface temperature early on. However, this error is only $O(\tau^{2})$. (iv) The full ODE given in equation (2.12) has been derived to improve upon the Myers model by accounting for the change in the sensible heat of the accumulating ice layer. Nevertheless, most surprisingly, its steady-state prediction is not as good as the Myers model. This indicates the cancelling effect of errors due to different approximations in the Myers model yielding very good overall limiting behaviours. The rime ice solution is meaningful only till the critical time $t_{c}$, when the rime ice surface temperature reaches the melting point. Any further droplet impaction at the ice surface will result in the formation of a water layer on top of the ice layer, leading to glaze ice formation. The transition time and the rime ice thickness at the end of the rime ice phase can be estimated using the Myers model as

**Table 2.** Short-time and steady-state asymptotic behaviour of the non-dimensional ice surface temperature predicted by the Myers model (*θ*_*M*,*I*_), the ODE obtained from the linear temperature profile (*θ*_*O*,*I*_) and the PDE obtained from the rigorous formulation (*θ*_*P*,*I*_).

		*θ_I_*(τ≪1)	$\theta_{I}(\tau \to \infty )$
*θ*_*M*,*I*_		Bτ(1−Aτ)	*B*/*A*
$\theta_{O,I}$	full ODE (2.12)	Bτ(1−Aτ)	*B*/(*A*−1/2)
	ignoring ∂/∂τ	Bτ(1−(A−1/2)τ)	*B*/(*A*−1/2)
$\theta_{P,I}$	full PDE (2.7)	Bτ(1−Aτ)	*B*/*A*
	ignoring ∂/∂τ	Bτ(1−(A−1/3)τ)	*B*/*A*


(2.14) $$\tau_{M,c}=\frac{1}{B-A}\;\text{ and }\;\frac{h_{M,t}}{L}=\frac{1}{B-A}.$$

Two different behaviours can be identified. If B>A, then there exists a positive critical time $t_{c}=\tau_{c}\alpha_{i}/{\dot{h}}_{im}^{2}$ when rime ice formation ends, with the corresponding rime ice thickness given by ${\dot{h}}_{im}\;t_{c}$. On the other hand, if B<A (indicating very small values of ${\dot{m}}_{im}$), then rime ice accretion proceeds indefinitely, as illustrated in figure 2. Physically, this can be interpreted as the ice accretion occurring so slowly that the heat gained from latent heat release and other mechanisms is easily balanced by heat loss, preventing the ice surface temperature from reaching the melting point.

The estimate given in equation (2.14), which is obtained using the Myers model, is only approximate. Nevertheless, when B>A, i.e. for conditions when rime ice transitions to glaze ice beyond a critical time $t_{c}$, ice surface temperature estimates for times greater than $t_{c}$ become irrelevant. In particular, for B≫A, the long-time asymptotic steady state given in table 2 is not relevant since it corresponds to ice surface temperatures larger than the melting point temperature, and thus the rime ice phase will be finished well before the asymptotic state is approached.

### (e) Numerical solution and comparison

**Figure 4.**
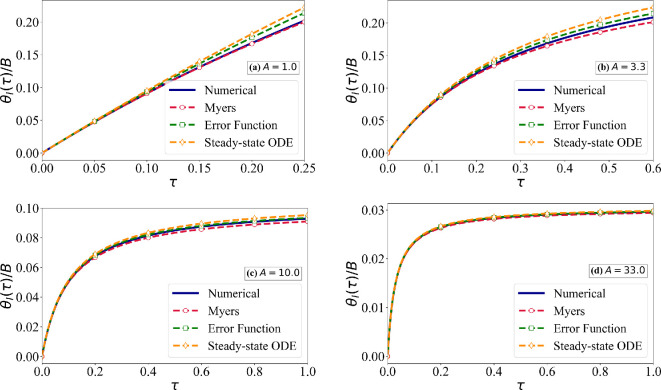
Comparison of the exact numerical solution of (2.7) for constant ice properties for four different values of *A* against (i) Myers model (2.11), (ii) error function solution (2.10) and (iii) steady-state solution of the ODE model (2.13).

We now numerically solve the PDE and boundary conditions given in equation (2.7) without any additional approximations. The numerical solution, along with the analytical solutions, linearly scales with B. Thus, it is sufficient to consider a plot of scaled ice surface temperature $\theta_{I}(\tau )/B$ versus τ for different values of A. Figure 4 shows results for A=1.0,3.3,10.0 and 33.0. Each frame shows (i) the exact numerical solution of equation (2.7), (ii) the Myers solution equation (2.11), (iii) the long-time approximation of the exact solution given in equation (2.10) and (iv) the long-time approximation of the ODE solution given in equation (2.13). We are interested only in the scaled ice surface temperature (i.e. the vertical axis) in the range $0\leq \theta_{I}/B\leq \text{min}\{1/B,1/A\}$. Based on the earlier estimate that the smallest possible value of B is O(5), the plots for A=1.0 and 3.3 are shown over the range τ≤0.25 and 0.6, since the approach to steady state is not relevant. In contrast, the plots for A=10.0 and 33.0 are presented over the range τ≤1, where the approach to steady state is relevant.

**Figure 5.**
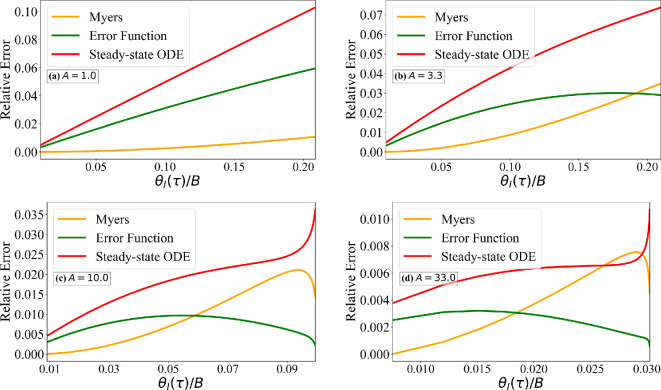
Comparison of the true relative error (as defined in (2.13)) in the critical time, computed from the exact numerical solution of (2.7) for four different values of A, with the predictions of (i) the Myers model (2.11), (ii) the error function solution (2.10) and (iii) the steady-state solution of the ODE model (2.13).


Several observations can be made. First, as discussed in table 2, the Myers model is quite good in matching the short-time initial rise and the asymptotic final value. At intermediate times, it slightly underpredicts the interface temperature. As a result, the transition time corresponding to the vertical axis being equal to 1/B will be slightly overpredicted by the Myers model. Correspondingly, the rime ice thickness at the critical time will be slightly overpredicted. Second, for small values of A, the numerical solution is quite close to the Myers solution, while for larger values of A, the numerical solution is better approximated by the error function solution given in equation (2.10). We note that the asymptotic value of the plots is given by $\theta_{I}(\tau \to \infty )/B\to \left(1/A\right)$. Overall, the error incurred in the Myers model is quite small. The critical time $\tau_{c}$ for the transition from rime to glaze ice corresponds to $\theta_{I}(\tau_{c})=1.0$. Thus, the critical time can be estimated from figure 4 by looking at the value of τ along the horizontal axis for a vertical axis value of 1/B. This result is presented in figure 5, where the relative error between the exact numerical value of critical time and its approximation by the analytical models is defined as

(2.15) $$\text{ relative error }=\frac{\left|\tau_{c,\text{ exact }}-\tau_{\text{approx }}\right|}{\tau_{c,\text{ exact }}}$$

is plotted as a function of $\theta_{I}/B$ for the four different values of A. Note that the maximum value of $\theta_{I}/B$ of interest is 1/B when B>A (corresponding to the critical time of transition from rime to glaze ice) and 1/A when B<A (where no critical time exists and rime ice continues to form without transitioning to glaze ice). For larger values of A, the results are presented for $0\leq \theta_{I}/B\leq 1/A$. For values of A slightly in excess or equal to one, the relative error in the Myers model is negligibly small. Nevertheless, it increases with time and reaches the largest value at the critical time when $\theta_{I}=1$. For larger values of A, when A>B, a critical time does not exist. The relative error is identically zero both at t=0 and as t→∞. In this limit, from figure 4, it can be seen that the Myers model prediction can be in error at intermediate times, with the peak error reaching about a few per cent. The maximum relative error occurs when B is small and A is of a similar magnitude. Nevertheless, the error in the Myers model remains small.

**Figure 6.**
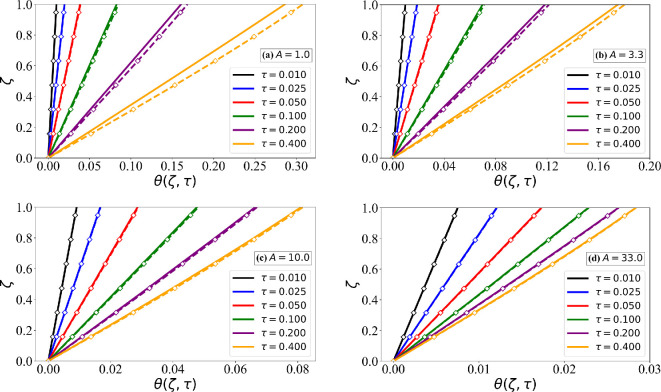
Plot of θ(ζ,τ) versus ζ for different values of A and τ using the exact numerical solution of (2.7)—solid lines. Also plotted as dashed lines are the error function profiles given in (2.10).

We now proceed to evaluate the validity of the linear thermal profile assumption used in the simpler models. In figure 6, we plot θ(ζ,τ) versus ζ as solid lines for the four different values of A and for different values of τ. The temperature profiles at early times τ<0.1 for A=1.0 show a small departure from the linear profile. Also plotted in figure 6 are the error function profiles given in equation (2.10). The early time deviation from the linear profile is well captured by the error function solution. For larger values of A>1 and at later times (τ>0.1), the thermal profiles become nearly linear, which agrees well with the accurate predictions of the Myers model. Although the error function solution also reduces to a near-linear profile for larger values of τ, it differs from the exact temperature profile at small values of A.

## 3. Rime ice formulation—variable ice properties

In this section, we extend the analysis to investigate the added effects of variable ice density, variable ice conductivity and sublimation from the ice surface. Variable ice property in the context of steady conduction has been studied assuming linear variation in ice density and conductivity across the ice layer [22]. Here we will first examine the nature of property variation to determine its functional dependence on z.

### (a) Ice density and thermal conductivity variation

Towards this end, we denote the time at which rime ice is deposited at any distance $z^{*}$ from the airfoil surface to be $t^{*}$. Let the ice surface temperature at this time of initial deposition be $T_{I}^{*}=T(z^{*},t^{*})$. For example, figure 3 shows ice deposition at $h_{j}$ at time $t_{j}$ with surface temperature $T_{Ij}$ for j=1,2,⋯. We then assume that (i) the ice density at $z^{*}$ depends only on the surface temperature $T_{I}^{*}$ at the time of deposition and (ii) the ice density at $z^{*}$ remains unchanged at all later times. As ice accretion continues and the ice layer thickness increases, the ice temperature at any location $z^{*}$ decreases. However, the ice density remains the same as that determined by $T_{I}^{*}$ of that elevation. The second condition arises from the reasonable assumption that the density of an ice layer, once formed, cannot change over time. These assumptions lead to a more complex density variation across the ice layer.

We now assume ice density to be a linear function of the interface temperature at the time of ice formation. With this assumption, ice density can be expressed as


(3.1) $$\rho_{i}=\rho_{i0}+\Delta_{\rho }\frac{\left(T_{I}-T_{s}\right)}{\left(T_{f}-T_{s}\right)},$$

where $\rho_{i0}$ is the rime ice density when it first forms on the airfoil surface at the airfoil temperature $T_{s}$ and $\Delta_{\rho }=\rho_{g}-\rho_{i0}$, where $\rho_{g}$ is the glaze ice density corresponding to the density at the melting temperature $T_{f}$. Thus, as $T_{I}$ varies from $T_{s}$ to $T_{f}$, ice density increases linearly from $\rho_{i0}$ to $\rho_{g}$.

Rime ice has a lower density than solid ice due to its porous structure. The air pockets that contribute to ice porosity also reduce its thermal conductivity. It can be shown that rime ice conductivity is linear in density and thus linear in ice surface temperature. This linear relation can be expressed as


(3.2) $$k_{i}=k_{i0}+\Delta_{k}\frac{\left(T_{I}-T_{s}\right)}{\left(T_{f}-T_{s}\right)},$$

where $k_{i0}$ is the conductivity of rime ice when it forms on the airfoil surface at $T_{s}$ and $\Delta_{k}=k_{g}-k_{i0}$, where $k_{g}$ is the glaze ice conductivity.

With variable ice properties, the length and time scales of the previous section must be modified in terms of their initial value as


(3.3) $$L=\frac{k_{i0}}{C_{p,i}{\dot{m}}_{im}}\;\text{ and }\;T=\frac{k_{i0}\rho_{i0}}{C_{p,i}{\dot{m}}_{im}^{2}}.$$

The non-dimensional z and time are then defined as


(3.4) $$\tilde{z}=\frac{z}{L}\;\text{ and }\;\tau =\frac{t}{T}.$$

We seek an asymptotic solution that is valid for small values of the non-dimensional time (i.e. for τ≪1). As in the previous section, we expand the non-dimensional ice temperature at the ice–air interface as


(3.5) $$\begin{array}{lcr} & \theta_{I}=\frac{\left(T_{I}-T_{s}\right)}{\left(T_{f}-T_{s}\right)}=\theta_{I1}\;\tau +\theta_{I2}\;\tau^{2}+\cdots \;, & \end{array}$$

which when substituted into equations (3.1) and (3.2) yields


(3.6) $$\text{ At ice–air interface: }\left\{ \begin{array}{l} \rho_{I}=\rho_{i0}\left[1+{\hat{\Delta }}_{\rho }\left(\theta_{I1}\tau +\theta_{I2}\tau^{2}+\cdots \right)\right]\\ k_{I}=k_{i0}\left[1+{\hat{\Delta }}_{k}\left(\theta_{I1}\tau +\theta_{I2}\tau^{2}+\cdots \right)\right], \end{array}\right.$$

where ${\hat{\Delta }}_{\rho }=\Delta_{\rho }/\rho_{i0}$ and ${\hat{\Delta }}_{k}=\Delta_{k}/k_{i0}$ are important parameters that measure the level of density and thermal conductivity variation across the rime ice layer compared to their initial value. As clearly indicated by the subscript ‘I’, the above-given expressions apply only to the density and thermal conductivity of the ice layer that forms at the surface at time τ.

In order to obtain the ice density and thermal conductivity as a function of $\tilde{z}$, we must first find the time of formation, which when substituted into equation (3.6) will yield the desired result. We now write the non-dimensional time of ice formation as an expansion in $\tilde{z}$ as


(3.7) $$\text{ At the time of formation: }\;\tau =\frac{t}{T}=a_{1}\tilde{z}+a_{2}{\tilde{z}}^{2}+\cdots .$$

Substituting this into equation (3.6), we obtain


(3.8) $$\text{ Within the ice layer: }\left\{ \begin{array}{l} \rho_{i}(\tilde{z})=\rho_{i0}[1+\underset{=\delta_{\rho 1}}{\underbrace{{\hat{\Delta }}_{\rho }\theta_{I1}a_{1}}}\tilde{z}+\underset{=\delta_{\rho 2}}{\underbrace{{\hat{\Delta }}_{\rho }\left(\theta_{I1}a_{2}+\theta_{I2}a_{1}^{2}\right)}}{\tilde{z}}^{2}+\cdots ]\\ k_{i}(\tilde{z})=k_{i0}[1+\underset{=\delta_{k1}}{\underbrace{{\hat{\Delta }}_{k}\theta_{I1}a_{1}}}\tilde{z}+\underset{=\delta_{k2}}{\underbrace{{\hat{\Delta }}_{k}\left(\theta_{I1}a_{2}+\theta_{I2}a_{1}^{2}\right)}}{\tilde{z}}^{2}+\cdots ]. \end{array}\right.$$

The above-given expressions can be used to evaluate ice density and thermal conductivity at any point $\tilde{z}$ within the ice layer. They involve expansion coefficients $\left\{\theta_{I1},\theta_{I2},\cdots \;\right\}$ and $\left\{a_{1},a_{2},\cdots \;\right\}$, which will be determined below as part of the solution. Once these coefficients are determined, the above-given expressions offer a physically meaningful description of the variable properties.

### (b) Mass balance with sublimation

We will rederive the mass balance equation (equation (2.4)) with the inclusion of mass removal from the ice surface through sublimation. Sublimation is driven by the partial pressure difference between the ice surface temperature and the far-field temperature. By locally linearizing the dependence of the partial pressure on temperature, the rate of mass removal per unit area of the ice surface per unit time can be expressed as ${\dot{m}}_{s}=\chi_{s}\;(T_{I}-T_{a})$, where $\chi_{s}$ is the mass sublimation coefficient. The temperature dependence can be non-dimensionalized and expressed in terms of the mass rate of impingement as


(3.9) $$\begin{array}{lcr} & {\dot{m}}_{s}={\dot{m}}_{im}\;{\hat{\chi }}_{s}\;(\theta_{I}-\theta_{a})\;, & \end{array}$$

where ${\hat{\chi }}_{s}=\chi_{s}(T_{f}-T_{s})/{\dot{m}}_{im}$ is the non-dimensional sublimation coefficient. Along with ${\hat{\Delta }}_{\rho }$ and ${\hat{\Delta }}_{k}$, ${\hat{\chi }}_{s}$ is an important quantity that will parameterize the importance of sublimation relative to the mass of ice accretion by droplet impingement.

The mass balance equation (equation (2.4)) is modified as


(3.10) $$\rho_{I}\frac{\text{d}h}{\text{d}t}={\dot{m}}_{im}-{\dot{m}}_{s}\;.$$

The following two important changes can be observed: (i) the sublimation mass rate is subtracted, and (ii) since ice density is not a constant, we now correctly use the ice density at the surface in the mass balance. The above mass balance is non-dimensionalized using the time and temperature scales. In addition, we substitute $\hat{h}=h/L$ as the non-dimensional rime ice thickness. Substituting equation (3.6) for $\rho_{I}$ and equation (3.9) for ${\dot{m}}_{s}$, we obtain


(3.11) $$\left[1+{\hat{\Delta }}_{\rho }\theta_{I}\right]\frac{\text{d}\hat{h}}{\text{d}\tau }=\left(1-{\hat{\chi }}_{s}(\theta_{I}-\theta_{a})\right)\;.$$

An asymptotic solution that is valid for τ≪1 can be obtained by expanding the non-dimensional ice thickness as $\hat{h}(\tau )={\hat{h}}_{1}\;\tau +{\hat{h}}_{2}\;\tau^{2}+\cdots$ and substituting equation (3.5) for $\theta_{I}$. We then obtain the following solution for the non-dimensional ice thickness as a function of non-dimensional time


(3.12) $$\begin{array}{lcr} & \hat{h}(\tau )=\underset{={\hat{h}}_{1}}{\underbrace{\left(1+{\hat{\chi }}_{s}\;\theta_{a}\right)}}\tau -\underset{={\hat{h}}_{2}}{\underbrace{\frac{1}{2}\theta_{I1}\left({\hat{\chi }}_{s}+{\hat{\Delta }}_{\rho }(1+{\hat{\chi }}_{s}\;\theta_{a})\right)}}\tau^{2}+O(\tau^{3})\;. & \end{array}$$

In the limit when ice density is a constant (i.e. ${\hat{\Delta }}_{\rho }=0$) and sublimation can be ignored (i.e. ${\hat{\chi }}_{s}=0$), we obtain the expected constant ice growth as $\hat{h}(\tau )=\tau$. To O(τ), the ice thickness is affected only by sublimation through the added contribution of ${\hat{\chi }}_{s}\;\theta_{a}\tau$ that appears in the ${\hat{h}}_{1}$ expression. If the airfoil and ambient temperatures are the same, then $\theta_{a}=0$, and the leading order effect of sublimation vanishes. If $T_{a}<T_{s}$, then $\theta_{a}$ is negative, and sublimation, as expected, decreases the rate of growth of ice thickness. Since rime ice density increases with z, ${\hat{\Delta }}_{\rho }$ is positive, and the $O(\tau^{2})$ effect of variable density is to decrease the ice thickness growth rate. In the above-given ice thickness solution, while ${\hat{\Delta }}_{\rho }$ and ${\hat{\chi }}_{s}$ are external inputs that parameterize the effects of variable density and sublimation, respectively, $\theta_{I1}$ is an internal input that can only be determined through further analysis of the energy equation.

### (c) Unsteady energy equation and its asymptotic solution

We start with the unsteady one-dimensional heat conduction equation given in equation (2.1) along with the boundary conditions given in equation (2.2). The differences now are (i) $\rho_{i}$ and $k_{i}$ are functions of z, (ii) the ice thickness is given by equation (3.12) and (iii) with non-zero sublimation, we redefine $A^{\prime }=q_{c}+q_{d}+q_{s}$, where $q_{s}$ accounts for heat loss through sublimation. We follow the steps of §2, by first transforming to the new variable ζ=z/h(t), which upon scaling with the length scale yields the relation $\tilde{z}=(z/L)=\zeta \;\hat{h}$. Then, we non-dimensionalize the governing equation to obtain


(3.13) $$\begin{array}{lcr} & (1+\delta_{\rho 1}\zeta \hat{h}+\delta_{\rho 2}\zeta^{2}{\hat{h}}^{2}+\cdots \;)\left({\hat{h}}^{2}\frac{\partial \theta }{\partial \tau }-\zeta \hat{h}\frac{\partial \hat{h}}{\partial \tau }\frac{\partial \theta }{\partial \zeta }\right)=\frac{\partial }{\partial \tau }\left((1+\delta_{k1}\zeta \hat{h}+\delta_{k2}\zeta^{2}{\hat{h}}^{2}+\cdots \;)\frac{\partial \theta }{\partial \zeta }\right)\;. & \end{array}$$

The above-given equation reduces to equation (2.7) in the limit when sublimation and variable properties are ignored (note that in this limit $\hat{h}=\tau$). The boundary condition on the airfoil surface and the Stefan condition at the ice surface can similarly be expressed as


(3.14) $$\theta (\zeta =0,\tau )=0,\;\text{ and }{\;\left(1+{\hat{\Delta }}_{k}\theta_{I}\right)\frac{\partial \theta }{\partial \zeta }|}_{\zeta =1}=\hat{h}\left(B-A\theta_{I}\right).$$

An asymptotic solution is obtained by expanding the ice layer temperature θ in a power series as given in equation (2.9). The steps involved are somewhat tedious, owing to the fact that the expansion of equation (3.12) must be substituted into equation (3.13). Furthermore, the unknown coefficients $\left\{\theta_{I1},\theta_{I2},\cdots \;\right\}$ (see equation (3.5)) and $\left\{a_{1},a_{2},\cdots \;\right\}$ (see equation (3.7)) that appear in $\delta_{\rho 1}$, $\delta_{k1}$, etc., must be simultaneously determined in a self-consistent manner. For example, the θ expansion must be equal to the $\theta_{I}$ expansion in the limit ζ=1. Carrying out these calculations, we obtain the final non-dimensional ice temperature distribution as


(3.15) $$\begin{array}{lcr} & \theta (\zeta ,\tau )=B\;{\hat{h}}_{1}\;\zeta \;\tau -\left(\left(A\;B\;{\hat{h}}_{1}^{2}+\frac{1}{2}B^{2}{\hat{h}}_{1}({\hat{\chi }}_{s}+{\hat{\Delta }}_{\rho }{\hat{h}}_{1})\right)\zeta +\frac{1}{2}{\hat{\Delta }}_{k}\;B^{2}\;{\hat{h}}_{1}^{2}\zeta^{2}\right)\tau^{2}+O(\tau^{3})\;. & \end{array}$$

Note that in the above equation, ${\hat{h}}_{1}=1+{\hat{\chi }}_{s}\theta_{a}$. With this, we have obtained an explicit expression for the time and depth dependence of the non-dimensional temperature within the ice layer. This solution depends on the non-dimensional parameters A and B that quantify heat loss and heat addition mechanisms at the ice surface. The solution also depends on the coefficients ${\hat{\Delta }}_{\rho }$, ${\hat{\Delta }}_{k}$ and ${\hat{\chi }}_{s}$ that parameterize density variation, thermal conductivity variation and sublimation, respectively.^1^

By setting ${\hat{\Delta }}_{\rho }={\hat{\Delta }}_{k}={\hat{\chi }}_{s}=0$, it can be verified that in the limit of constant ice properties and no sublimation, the above-given solution is the same as that given in equation (2.10). Also, even higher-order terms can be systematically derived. However, such effort is not necessary, as we will see below, the above-given $O(\tau^{2})$ expansion is sufficient to investigate the effects of variable properties and sublimation.

The above-given expansion can be used to evaluate the short-time evolution of the interface temperature. The long-time result for τ→∞ can also be obtained from the governing equation (equation (3.13)) and the boundary conditions (equation (3.14)). In fact, the long-time solutions are dictated by the Stefan condition and are unaffected by the variable properties. As a result, the steady-state value remains the same as that for constant properties decided by the balance between heat addition and heat loss processes at the ice surface. These short- and long-time results for the ice surface temperatures are


(3.16) $$\begin{array}{lcr} & \theta_{I}(\tau )=B\;{\hat{h}}_{1}\;\tau -\left(A\;B\;{\hat{h}}_{1}^{2}+\frac{1}{2}B^{2}{\hat{h}}_{1}({\hat{\chi }}_{s}+({\hat{\Delta }}_{\rho }+{\hat{\Delta }}_{k}){\hat{h}}_{1})\right)\tau^{2}\;,\;\text{for}\;\;\tau \ll 1\;. & \end{array}$$

### (d) Quantitative estimates of ${\hat{\Delta }}_{\rho },{\hat{\Delta }}_{k}$ and ${\hat{\chi }}_{s}$

A quantitative measure of variable properties can be obtained with a concrete example. As shown in [22,23], rime ice properties somewhat depend on impingement velocity and surface temperature. Based on their results, we let icing conditions be such that the initial ice density and conductivity are $\rho_{i0}=860$ kg m^−3^ and $k_{i0}=1.8$ W (m^−1^-K^−1^). The corresponding values for glaze ice are well-established to be a constant [11] and can be taken to be $\rho_{g}=917$ kg m^−3^ and $k_{g}=2.22$ W (m^−1^-K^−1^). This yields ${\hat{\Delta }}_{\rho }=0.066$ and ${\hat{\Delta }}_{k}=0.233$. As the initial ice density and conductivity change, the non-dimensional coefficients will change somewhat. However, the above-given values are sufficient to illustrate the effect of variable properties.

**Figure 7.**
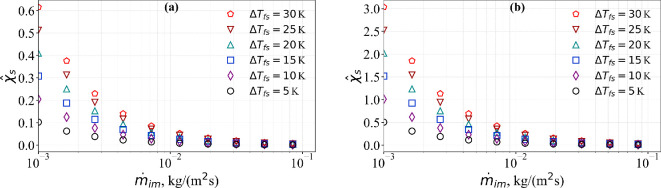
(a) Plot of ${\hat{\chi }}_{s}$ versus mass impingement rate ${\dot{m}}_{im}$ for (a) case 1 and (b) case 2 for different $\Delta T_{fs}$.

Using well-established expressions for sublimation mass [11], the sublimation coefficient can be expressed as


(3.17) $$\begin{array}{lcr} & {\hat{\chi }}_{s}=\frac{\chi_{s}(T_{f}-T_{s})}{{\dot{m}}_{im}}=\frac{6.22\;h_{c}\;e_{0}}{C_{P,a}\;p_{0}\;Le^{2/3}}\frac{\left(T_{f}-T_{s}\right)}{{\dot{m}}_{im}}\;, & \end{array}$$

where $h_{c}$ is the convective heat transfer coefficient at the ice surface, $e_{0}=27.3$ is the vapour pressure constant, $C_{P,a}$ is the specific heat of air, $p_{0}$ is the ambient pressure and Le is the Lewis number. While all other values in the above-given equation are reasonably known, ${\hat{\chi }}_{s}$ can take large or small values depending on the mass rate of droplet impingement. Again we consider the low free-stream velocity case 1 and high free-stream velocity case 2 discussed in figure 2. The corresponding convective heat transfer coefficients can be taken to be $h_{c}=70$ and 345 W (m^−2^·K^−1^), respectively [14,24]. In addition, following the recommendations of Myers [11], we take $e_{0}=27.3$, $C_{P,a}=1014$ J (kg^−1^-K^−1^), *p*_0_ = 101325 Pa and Le=13.4. With these values, we calculate ${\hat{\chi }}_{s}$ for varying values of droplet mass impingement rate and $\Delta T_{fs}$. The results are presented in figure 7. From the figure, it is clear that ${\hat{\chi }}_{s}$ decreases with increasing ${\dot{m}}_{im}$ and decreasing $\Delta T_{fs}$. Furthermore, the values of ${\hat{\chi }}_{s}$ are substantially higher in case 2 than in case 1. Based on this figure, we will consider ${\hat{\chi }}_{s}=0.1$ and 1.0 as two representative values for further discussion, and we have already considered the limit of zero sublimation (i.e. ${\hat{\chi }}_{s}=0.0$).

Using equation (3.16), we can now estimate the impact of variable properties and sublimation on ice surface temperature. First, it can be noted that the effect on short-time ice surface temperature is only at $O(\tau^{2})$. The percentile changes in the short-time $O(\tau^{2})$ behaviour due to density variation, conductivity variation and sublimation can be estimated as $(B/2A){\hat{\Delta }}_{\rho }$, $(B/2A){\hat{\Delta }}_{k}$ and $\left(B/2A)({\hat{\chi }}_{s}/{\hat{h}}_{1}\right)$, respectively. For case 1 and case 2, for varying values of ${\dot{m}}_{im}$ and $\Delta T_{fs}$, we can use figures 2 and 7 for A, B and ${\hat{\chi }}_{s}$ and then use them to obtain quantitative estimates of the importance of variable properties and sublimation. In general, at short times, we see that larger values of B/A will increase the importance of variable properties and sublimation. The impact over the entire time range of interest will be evaluated numerically in a subsequent subsection.

### (e) Different behaviours of rime ice accretion

In the absence of sublimation (i.e. ${\hat{\chi }}_{s}=0$), we earlier observed two different behaviours of rime ice accretion. For B>A, the rime ice growth ended at the critical time $t_{c}$, and for B<A, rime ice continued to grow indefinitely without the ice surface reaching the melting point, i.e. without transitioning to glaze ice. With the inclusion of sublimation, three different behaviours can be identified. To arrive at this conclusion, we must recognize the following three constraints on the interface temperature: (i) rime ice requires $\theta_{I}\leq 1$; otherwise, the ice surface temperature will exceed the melting point. (ii) From the right-hand side of the mass balance (equation (3.11)), we require $\theta_{I}\leq (1+{\hat{\chi }}_{s}\theta_{a})/{\hat{\chi }}_{s}$; otherwise, the ice thickness will decrease. (iii) From the right-hand side of the Stefan condition (equation (3.14)), we require $\theta_{I}\leq B/A$; otherwise, the direction of heat conduction within the ice layer will reverse.

At the beginning when icing starts, $\theta_{I}=0$, and as ice accretion proceeds, $\theta_{I}$ increases. Rime ice accretion stops when $\theta_{I}$ reaches $\text{min}\{1,(1+{\hat{\chi }}_{s}\theta_{a})/{\hat{\chi }}_{s},B/A\}$. The three minimum possibilities are due to reaching the critical time for rime ice accretion, or reaching terminal ice thickness, or heat loss equalling the heat addition at the ice surface. Whichever condition is reached first will decide the ice surface temperature at the end of the ice phase. Based on this, the following three behaviours can be identified:

— *R1—Permanent rime ice phase of finite thickness*. Under the twin conditions of $(1+{\hat{\chi }}_{s}\theta_{a})/{\hat{\chi }}_{s}<\left\{1,B/A\right\}$, rime ice accretion will proceed forever, with the ice layer thickness approaching a constant value and the ice surface temperature remaining below the melting point. In this situation, strong sublimation governs the mass balance, which determines the final ice surface temperature and, consequently, the ultimate finite ice layer thickness as
(3.18) $$\theta_{I,\infty }=\frac{1+{\hat{\chi }}_{s}\theta_{a}}{{\hat{\chi }}_{s}}\;\text{ and }\;{\hat{h}}_{\infty }=\frac{\left({\hat{\chi }}_{s}+{\hat{\Delta }}_{k}\left(1+{\hat{\chi }}_{s}\theta_{a}\right)\right)\left(1+{\hat{\chi }}_{s}\theta_{a}\right)}{{\hat{\chi }}_{s}\left(B{\hat{\chi }}_{s}-A\left(1+{\hat{\chi }}_{s}\theta_{a}\right)\right)},$$
  Thus, mass loss by sublimation exactly balances mass addition by impaction to reach a finite thickness and an ice surface temperature below the melting point.
— *R2—Permanent rime ice phase of growing thickness*. Under the twin conditions of $B/A<\left\{1,(1+{\hat{\chi }}_{s}\theta_{a})/{\hat{\chi }}_{s}\right\}$, rime ice accretion will proceed for a long time, with the ice layer thickness growing indefinitely, while the ice surface temperature approaches a constant value below the melting point. In this situation, the thermal balance at the ice surface is achieved as heat addition and cooling cancel each other out, while the contribution from conduction vanishes. The corresponding ice surface temperature and the ice thickness growth rates are given by
(3.19) $$\theta_{I,\infty }=\frac{B}{A}\;\text{ and }{\;\frac{\text{d}\hat{h}}{\text{d}\tau }|}_{\infty }=\frac{\left(1-{\hat{\chi }}_{s}\left(B/A-\theta_{a}\right)\right)}{1+{\hat{\Delta }}_{\rho }(B/A)}.$$
  This was one of the two behaviours that we encountered earlier in the absence of sublimation.
— *R3—Transition to glaze ice in finite time*. Under the twin conditions of $1<\left\{B/A,(1+{\hat{\chi }}_{s}\theta_{a})/{\hat{\chi }}_{s}\right\}$, rime ice accretion will stop at a critical time $t_{c}$ when the ice surface temperature first reaches $\theta_{I}=1$. Neither mass balance between mass addition by droplet impaction and mass removal by sublimation nor thermal balance between heating and cooling at the ice surface will be achieved by this time. The nature of the steady-state solution (whether ice thickness goes to a finite value or grows forever) depends on which of B/A or $(1+{\hat{\chi }}_{s}\;\theta_{a})/{\hat{\chi }}_{s}$ is smaller. Nevertheless, this distinction of the steady state is not of interest since the rime ice phase will be over before the steady state is approached.

**Figure 8.**
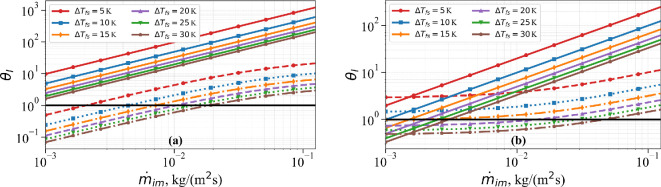
Regime map showing rime ice regime as a function of ${\dot{m}}_{im}$ and $\Delta T_{fs}$ for (a) case 1 and (b) case 2.

The results presented in figures 2 and 7 and similar ones calculated for $\theta_{a}\neq 0$ can be used to calculate B/A and $\left(1+{\hat{\chi }}_{s}\theta_{a})/{\hat{\chi }}_{s}\right\}$ and compare them with 1. Based on this comparison, for varying values of ${\dot{m}}_{im}$ and $\Delta T_{fs}$, we can identify the rime ice accretion regime to be R1, R2 or R3. This was done for small free-stream conditions of case 1 and high free-stream conditions of case 2, and the results are presented in figure 8, for the limit $\theta_{a}=0$. The horizontal black line corresponds to $\theta_{I}=1$, the dashed lines correspond to B/A and the solid lines correspond to $1/{\hat{\chi }}_{s}$. For any combination of ${\dot{m}}_{im}$ and $\Delta T_{fs}$, the lowest value of $\theta_{I}$ determines the rime ice regime.

Under case 1, we observe R1 to never occur. Regime R2, corresponding to permanent rime ice with ever-growing thickness, occurs for small ${\dot{m}}_{im}$, transitioning to regime R3 when rime ice transitions to glaze ice for larger ${\dot{m}}_{im}$. The transition value of ${\dot{m}}_{im}$ increases with increasing $\Delta T_{fs}$. This trend is consistent with the fact that at low mass impingement rates, the surface cooling effect that is primarily independent of ${\dot{m}}_{im}$ can easily balance the heating effect to reach a terminal ice surface temperature below the melting point. At a lower temperature difference ($\Delta T_{fs}$), the cooling effect decreases, and thus the transition value of ${\dot{m}}_{im}$ decreases. In case 2, at very low values of ${\dot{m}}_{im}$ and very large $\Delta T_{fs}$, we observe the possibility of permanent rime ice of finite thickness (regime R1). Also, over the range of ${\dot{m}}_{im}$ considered, for $\Delta T_{fs}<10$ K, rime ice always transitions to glaze ice, and this conclusion is consistent with the notion that only at ambient temperatures substantially lower than the melting point does significant rime ice form. The change from regime R2 to regime R3 is similar to that for case 1 but occurs at a higher value of ${\dot{m}}_{im}$.

### (f) Myers model with sublimation

Myers’ formulation [11] given in equation (2.11) changes in the presence of sublimation. The change is primarily in the ice thickness, whose growth rate decreases due to sublimation, which in turn affects the time evolution of the ice surface temperature. In the present notation, the Myers model with sublimation can be expressed as


(3.20) $$\frac{\text{d}{\hat{h}}_{M}(\tau )}{\text{d}\tau }=\left(1-{\hat{\chi }}_{s}\left(\theta_{I,M}-\theta_{a}\right)\right)\;\text{ and }\;\theta_{I,M}(\tau )=\frac{B{\hat{h}}_{M}}{1+A{\hat{h}}_{M}}.$$

The above-given ODE can be numerically solved to obtain the non-dimensional ice layer thickness and ice surface temperature as functions of non-dimensional time.

We now obtain the asymptotic solution for comparison with those of the complete equations. The non-dimensional ice height is given by


(3.21) $$\begin{array}{lcr} & {\hat{h}}_{M}(\tau )=\underset{={\hat{h}}_{1}}{\underbrace{\left(1+{\hat{\chi }}_{s}\;\theta_{a}\right)}}\tau -\frac{B}{2}{\hat{\chi }}_{s}(1+{\hat{\chi }}_{s}\;\theta_{a})\tau^{2}+O(\tau^{3})\;, & \end{array}$$

which equals the exact solution (equation (3.12)) in the limit of constant ice properties. The corresponding short- and long-time solutions for the non-dimensional ice surface temperature are given by


(3.22) $$\theta_{I,M}(\tau )=\left\{ \begin{array}{ll} B{\hat{h}}_{1}\tau -\left(AB{\hat{h}}_{1}^{2}+\frac{1}{2}B^{2}{\hat{h}}_{1}{\hat{\chi }}_{s}\right)\tau^{2}, & \text{ if }\tau \ll 1\\ \min\left\{\frac{B}{A},\frac{\left(1+{\hat{\chi }}_{s}\theta_{a}\right)}{{\hat{\chi }}_{s}}\right\} & \text{ if }\tau \to \infty . \end{array}\right.$$

Comparing the above-given equation with equation (3.16), it can be confirmed that the two are the same in the limit ${\hat{\Delta }}_{\rho }={\hat{\Delta }}_{k}=0$. In other words, the Myers solution accurately captures the effect of sublimation both in the short- and long-time limits. Again, we emphasize that the long-time solution is of relevance only in the limit $\text{min}\left\{B/A\;,\left(1+{\hat{\chi }}_{s}\;\theta_{a}\right)/{\hat{\chi }}_{s}\right\}<1$. If this condition is met, then, if $\left(1+{\hat{\chi }}_{s}\;\theta_{a}\right)/{\hat{\chi }}_{s}$ is smaller than blue B/A, both the ice thickness and ice surface temperature will reach their terminal values (regime R1). On the other hand, if B/A is smaller than $\left(1+{\hat{\chi }}_{s}\;\theta_{a}\right)/{\hat{\chi }}_{s}$, then the ice surface temperature would reach its terminal value below the melting point while continuing to grow in thickness indefinitely (regime R2). Otherwise, rime ice will transition to glaze ice well before the long-time solution is reached (regime R3). These behaviours are the same as the exact solution.

### (g) A modified Myers model

The above-given Myers solution does not account for the effects of variable properties. In this section, we examine the possibility of modifying the Myers model to incorporate the effects of variable density and thermal conductivity in a simple manner. By comparing equation (3.21) with equation (3.12), we note that the effect of variable ice density slows down the rate of increase in ice thickness, due to increasing ice surface density. This effect of time-dependent ice surface density can be easily incorporated into the Myers model. In fact, it can be shown that the appearance of ${\hat{\Delta }}_{\rho }$ in the short-time ice surface temperature given in equation (3.16) is also due to this time variation of ice surface density. As a result, by modifying the ice thickness equation, we can incorporate the effect of variable density not only in the prediction of ice thickness evolution but also in the ice surface temperature evolution.

To incorporate the effect of variable conductivity, from a comparison of the short-time behaviour given in equations (3.22) and (3.16), we observe that the effect of ${\hat{\Delta }}_{k}$ can be included by redefining the heat loss coefficient as $A\to A+\frac{B}{2}{\hat{\Delta }}_{k}$. In other words, if A in the Myers model (equation (3.20)) was replaced with the above-redefined heat loss coefficient, the short-time solutions given in equation (3.22) will correctly recover the short-time asymptotic results given in equation (3.16). On the other hand, the long-time behaviour is correctly captured, and there is no need to redefine A for larger values of τ.

Based on the above-given considerations and by matching the numerical results to be discussed below, we propose the following MM model:


(3.23) $$\text{ Modified Myers model: }\left\{ \begin{array}{l} \left(1+{\hat{\Delta }}_{\rho }\theta_{I,\text{MM}}\right)\frac{\text{d}{\hat{h}}_{\text{MM}}(\tau )}{\text{d}\tau }=\left(1-{\hat{\chi }}_{s}\left(\theta_{I,\text{MM}}-\theta_{a}\right)\right)\\ \theta_{I,\text{MM}}(\tau )=\frac{B{\hat{h}}_{\text{MM}}+\alpha \;B{\hat{h}}_{\text{MM}}^{2}}{1+A_{ef}{\hat{h}}_{\text{MM}}+\alpha \;\max\left\{A,\frac{B{\hat{\chi }}_{s}}{\left(1+{\hat{\chi }}_{s}\theta_{a}\right)}\right\}{\hat{h}}_{\text{MM}}^{2}}, \end{array}\right.$$

where the effective heat loss coefficient is defined as


(3.24) $$\begin{array}{lcr} & A_{ef}=A+\frac{B}{2}{\hat{\Delta }}_{k}\;, & \end{array}$$

where we have introduced quadratic terms in the numerator and the denominator of the energy equation with a weighting coefficient whose value has been empirically determined as α=(0.2+0.05B)(1+tanh(0.6277(ln⁡(A)−ln⁡(2+0.5B)))). Asymptotic short-time and long-time solutions of the above-given equations can be obtained. The $O(\tau^{2})$ solution without the quadratic addition will match the exact solution given in equation (3.16) with the use of $A_{ef}$. Although the addition of the quadratic term formally alters the $O(\tau^{2})$ term, it has been chosen to optimally capture the effect of higher-order terms at intermediate times. The long-time behaviour is dictated by the quadratic term, which ensures the recovery of the desired limit of $\theta_{I,\text{MM}}\to B/A$ as τ→∞. The value of α becomes zero for small values of A where only the short-time behaviour is of importance until the critical time of rime ice. For larger values of A, α→0.512, and the exact behaviour is well recovered, as will be seen below. The above-given modification is not unique. There are other empirical modifications that can also be used to merge these two limits. Here, we have chosen the quadratic correction for its simplicity.

In essence, the MM model has been designed to give the correct limiting behaviours as well as match the entire solution. It should be stressed that in the limit A≪B, the critical time is reached well before the steady state, and therefore the long-time solution is not of direct relevance. Nevertheless, imposing this limiting behaviour is useful for constraining the solution at intermediate times. Certainly, for A⪆B, imposing the long-time constraint is important. It follows from equation (3.16) that the effects of variable density and thermal conductivity are to increase the effective value of A. As can be seen from figure 4, this has the effect of increasing the critical time at which ice surface temperature reaches $T_{f}$ and rime ice transitions to glaze ice. In the case of variable conductivity, the increase in thermal conductivity of ice above the initial value of $k_{i0}$ contributes to enhanced heat loss and a higher value of A. The effect of variable density is similar. The increase in ice density beyond its initial value, $\rho_{i0}$, reduces the rate of increase in ice thickness, which in turn enhances heat loss. Sublimation also contributes similarly by increasing heat loss.

### (h) Numerical evaluation and comparison

In this subsection, we solve the following mass and energy balance equations numerically without assuming power-law expansions for the ice properties:


(3.25) $$\begin{array}{lcr} & (1+{\hat{\Delta }}_{\rho }\theta_{I})\frac{d\hat{h}}{d\tau }=(1-{\hat{\chi }}_{s}(\theta_{I}-\theta_{a}))\;, & \end{array}$$

(3.26) $$\begin{array}{lcr} & (1+{\hat{\Delta }}_{\rho }\theta^{*})\left({\hat{h}}^{2}\frac{\partial \theta }{\partial \tau }-\zeta \hat{h}\frac{\partial \hat{h}}{\partial \tau }\frac{\partial \theta }{\partial \zeta }\right)=\frac{\partial }{\partial \tau }\left((1+{\hat{\Delta }}_{k}\theta^{*})\frac{\partial \theta }{\partial \zeta }\right)\;. & \end{array}$$

In equations (3.25) and (3.26), $\left(1+{\hat{\Delta }}_{\rho }\theta_{I}\right)$ corresponds to the density at the interface normalized by the initial value of $\rho_{i0}$, whereas $\left(1+{\hat{\Delta }}_{\rho }\theta^{*}\right)$ corresponds to the normalized density of the ice layer at ζ and τ, whose value remains the same as when the ice layer formed. Similarly, $\left(1+{\hat{\Delta }}_{k}\theta^{*}\right)$ corresponds to the normalized conductivity of the ice layer at ζ and τ. Again, we assume the ice density and thermal conductivity to linearly depend on the temperature at formation as given in equation (3.1).

**Figure 9.**
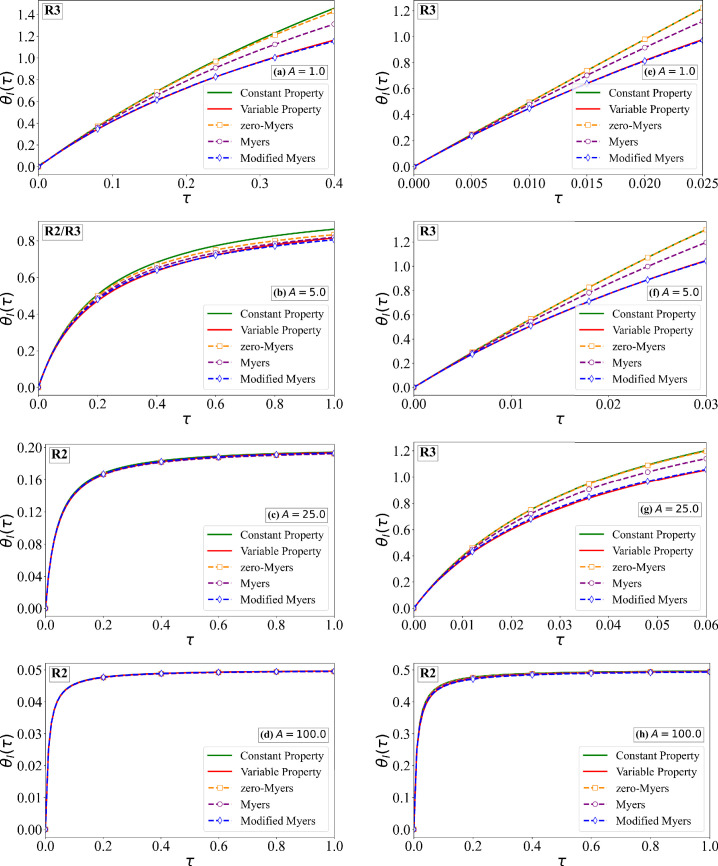
Non-dimensional surface temperature as a function of time for ${\hat{\chi }}_{s}$ = 0.15. The variable property numerical solution of (3.26) is compared against: (i) the constant property solution (2.7), (ii) the zero-Myers model (2.11) (ignoring sublimation and variable properties), (iii) the Myers model (3.20) (with sublimation) and (iv) the MM model (3.23) (with sublimation and variable properties). Results are shown for four different values of *A*, where the left column corresponds to *B* = 5.0 and the right column to *B* = 50.0.

**Figure 10.**
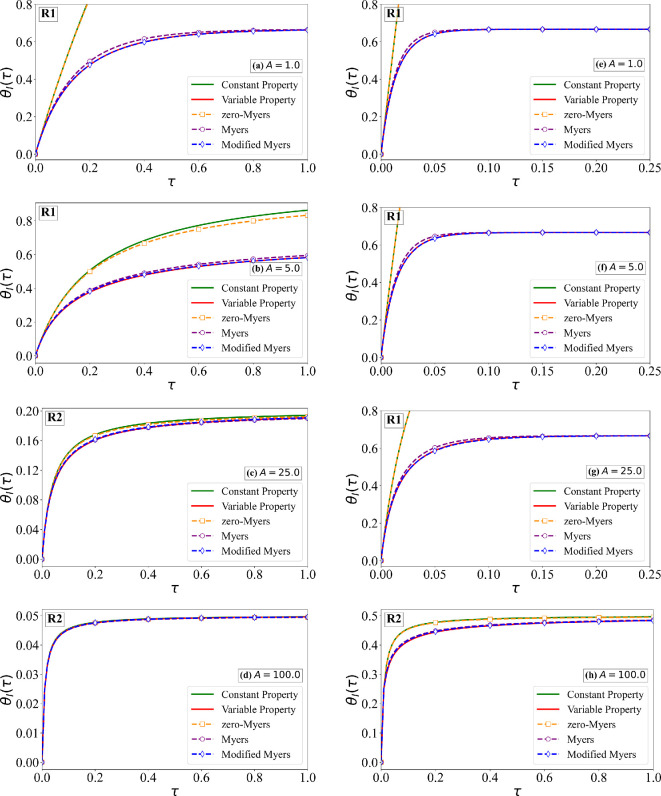
Non-dimensional surface temperature as a function of time for ${\hat{\chi }}_{s}$ = 1.50. The variable property numerical solution of (3.26) is compared against: (i) the constant property solution (2.7), (ii) the zero-Myers model (2.11) (ignoring sublimation and variable properties), (iii) the Myers model (3.20) (with sublimation) and (iv) the MM model (3.23) (with sublimation and variable properties). Results are shown for four different values of A, where the left column corresponds to B = 5.0 and the right column to B = 50.0.

**Figure 11.**
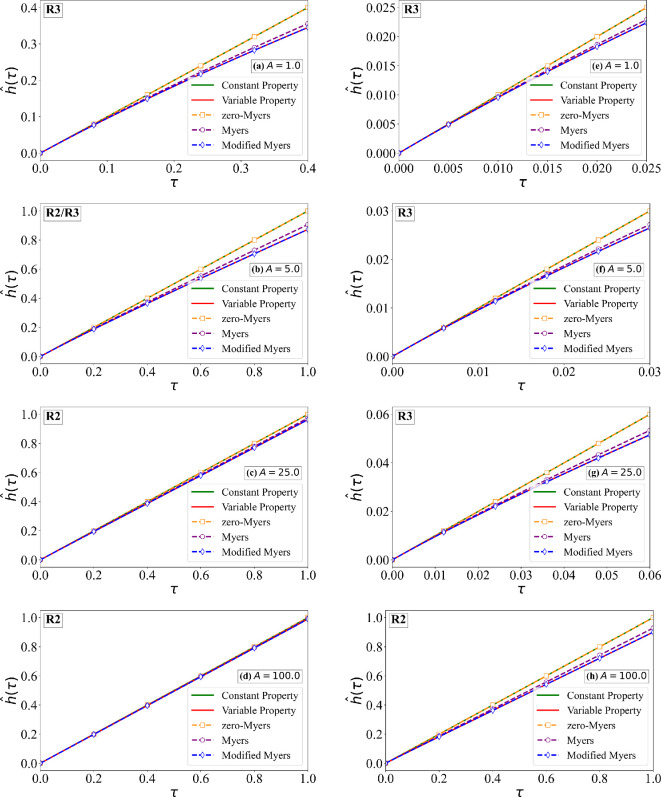
Non-dimensional ice thickness as a function of time for ${\hat{\chi }}_{s}$ = 0.15. The variable property numerical solution of (3.26) is compared against: (i) the constant property solution (2.7), (ii) the zero-Myers model (2.11) (ignoring sublimation and variable properties), (iii) the Myers model (3.20) (with sublimation) and (iv) the MM model (3.23) (with sublimation and variable properties). Results are shown for four different values of A, where the left column corresponds to B = 5.0 and the right column to B = 50.0.

**Figure 12.**
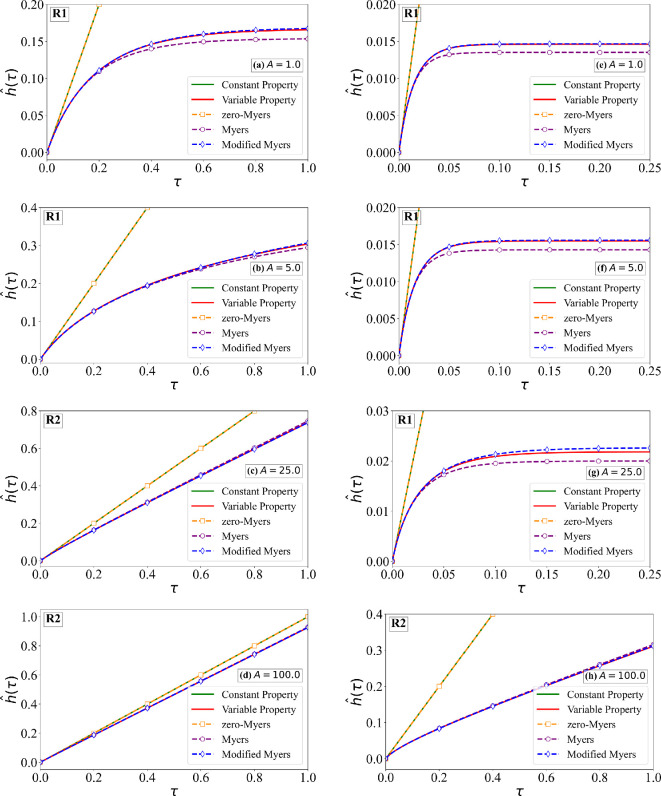
Non-dimensional ice thickness as a function of time with ${\hat{\chi }}_{s}$ = 1.50. The variable property numerical solution of (3.26) is compared against: (i) the constant property solution (2.7), (ii) the zero-Myers model (2.11) (ignoring sublimation and variable properties), (iii) the Myers model ((3.20)) (with sublimation) and (iv) the MM model (3.23) (with sublimation and variable properties). Results are shown for four different values of A, where the left column corresponds to B = 5.0 and the right column to B = 50.0.

As an example, we consider the cases of B=5 and 500, A=1,5,25 and 100, along with the earlier estimated values of ${\hat{\Delta }}_{\rho }=0.066$ and ${\hat{\Delta }}_{k}=0.233$. Two different values of the sublimation coefficient ${\hat{\chi }}_{s}=0.15$ and 1.5 are considered. From figures 2 and 7, we can find the values of physical quantities, such as the rate of droplet mass impingement, ambient temperature relative to the melting point and free-stream velocity, which correspond to these choices of non-dimensional quantities. Furthermore, with these choices, rime ice behaviour in all three regimes, R1, R2 and R3, is considered and is marked in each frame. The numerical solutions of the non-dimensional ice surface temperature for ${\hat{\chi }}_{s}=0.15$ and 1.5 are shown in figures 9 and 10. The corresponding estimates for the non-dimensional ice thickness are shown in figures 11 and 12, respectively. In the aforementioned four figures, ‘Constant Property’ corresponds to the earlier result presented in figure 4 for constant properties and zero sublimation (green line). ‘Variable Property’ corresponds to numerical results with variable properties and sublimation obtained by solving equations (3.25) and (3.26) (red line). The difference between these two results signifies the effect of variable properties and sublimation. From the figures, it is clear that the effects are significant except when A≫B. The difference particularly increases with increasing the sublimation coefficient.

Also shown in the figures are the following three different predictions: (i) ‘zero-Myers’ corresponds to the Myers model ignoring sublimation and variable properties (see equation (2.11)—plotted as orange square), (ii) ‘Myers’ corresponds to the Myers model with sublimation (see equation (3.20)—plotted as purple circle) and (iii) ‘Modified Myers’ corresponds to the MM model with all the effects included (see equation (3.23)—plotted as blue diamond). First and foremost, the MM model is in good agreement with the variable property numerical results, in terms of the ice surface temperature, since it was designed to achieve this result. The error in the prediction of ice surface temperature by the Myers model is the largest in regime R1, where the error increases with increasing values of B/A and $1/{\hat{\chi }}_{s}$. For example, for the case of B=50, A=1 and ${\hat{\chi }}_{s}=0.15$, the error in the prediction of critical time $\tau_{c}$ is about 20%. This error is primarily due to the neglect of variable properties in the Myers model, and therefore the error will be smaller for smaller values of ${\hat{\Delta }}_{\rho }$ and ${\hat{\Delta }}_{k}$. On the other hand, for even larger values of B/A and $1/{\hat{\chi }}_{s}$, the error will be higher. For smaller values of B/A and $1/{\hat{\chi }}_{s}$, the Myers model is reasonably accurate in its prediction of $\theta_{I}$.

The error in the prediction of ice thickness by the Myers model is generally small. Nevertheless, the trend is similar to that for $\theta_{I}$. The error in the prediction of ice thickness by the Myers model is the largest in regime R1, with the error increasing with increasing values of B/A and $1/{\hat{\chi }}_{s}$. The MM model is needed if one wants to compute ice surface temperature accurately. As to be expected, the Myers model without the effect of sublimation incurs much larger errors both in the ice thickness and ice surface temperature predictions.

### (i) Depth-dependent properties

**Figure 13.**
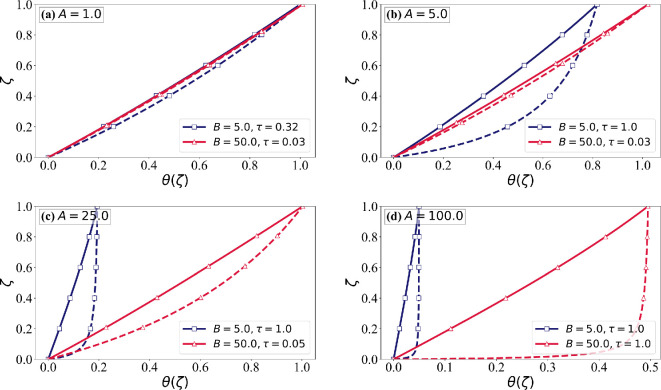
Temperature distribution, θ(ζ), as a function of the non-dimensional ice thickness (ζ) for different values of AandB, with ${\hat{\chi }}_{s}$ = 0.15. The solid lines correspond to the temperature distribution at time τ≤1.0 over the ice layer thickness ζ, while the dashed lines represent the formation temperature $\theta^{*}(\zeta )$. The red lines correspond to B = 50.0, while the dark blue lines represent B = 5.0. Each subplot corresponds to a different value of A: (a) *A* = 1.0, (b) *A* = 5.0, (c) *A* = 25.0 and (d) *A* = 100.0.

**Figure 14.**
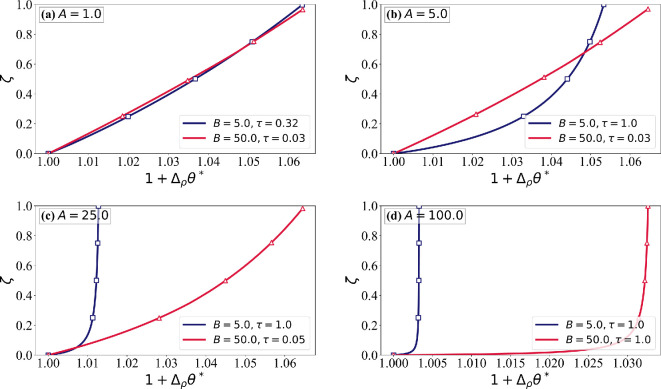
Normalized density variations ($1+\Delta_{\rho }\theta^{*}$) as a function of the non-dimensional ice thickness (ζ) for different values of A and B, with ${\hat{\chi }}_{s}$ = 0.15. The red line corresponds to B = 50.0, while the dark blue line represents B = 5.0. Each subplot shows results for different values of A: (a) *A* = 1.0, (b) *A* = 5.0, (c) *A* = 25.0 and (d) *A* = 100.0. The plots illustrate how density variations evolve with increasing ζ, highlighting the influence of B on profile shape and growth.

**Figure 15.**
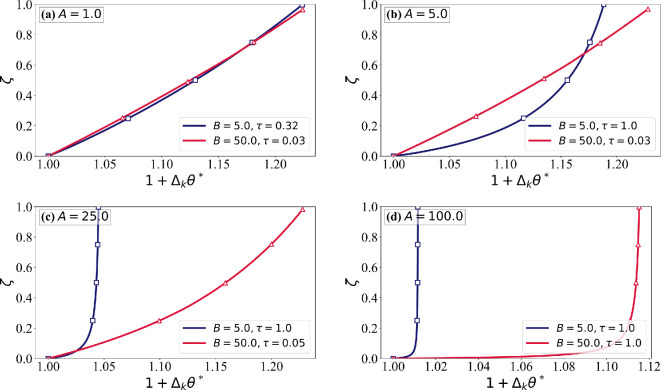
Normalized thermal conductivity variations ($1+\Delta_{k}\theta^{*}$) as a function of the non-dimensional ice thickness (*ζ*) for different values of A and B, with ${\hat{\chi }}_{s}$ = 0.15. The red line corresponds to B = 50.0, while the dark blue line represents *B* = 5.0. Each subplot shows results for different values of A: (a) *A* = 1.0, (b) *A* = 5.0, (c) *A* = 25.0 and (d) *A* = 100.0. The profiles resemble normalized density variations as both thermal conductivity and density depend on the formation temperature field $\theta^{*}$ governing phase change.

The two key assumptions often made in icing models are constant (depth-independent) ice properties and as a result linear temperature variation within the rime ice layer. The sensitivity to these assumptions will be examined. The depth-dependent evolution of ice properties is described by the ice formation temperature $\theta^{*}(\zeta )$. In figures 13–15, depth variation of the ice temperature and properties are plotted at τ= min $\left(\tau_{c},1.0\right)$*,* ensuring that the ice surface temperature does not exceed the critical threshold while maintaining consistency across different cases. In figure 13, the solid lines represent the ice temperature θ(ζ), while the dashed lines show the formation temperature $\theta^{*}(\zeta )$. As expected, the formation temperature of an ice layer is always higher than its temperature at a later time. The deviation between θ and $\theta^{*}$ reflects the role of conduction in redistributing heat within the ice layer as described earlier in figure 3. For low values of A, θ closely follows $\theta^{*}$, indicating a near-linear increase in the formation temperature. At high A, the formation temperature increases more rapidly initially and the increase slows down as rime ice accumulates. This increases the deviation between θ and $\theta^{*}$, particularly near the airfoil surface. The influence of B is significant. Lower B produces a steeper change in the formation temperature close to the airfoil surface.

Figures 14 and 15 illustrate the variations in normalized density and thermal conductivity as functions of the ice thickness ζ, highlighting the role of thermal and mass transport mechanisms. Both properties depend on $\theta^{*}$. At low A, we observe near-linear variations, whereas at high A, sharp gradients in ice properties are observed near the airfoil surface. The effect of B is also evident. These results confirm that A controls conduction-driven thermal smoothing, while B influences mass deposition and phase transition efficiency, shaping the depth dependence of the formation temperature, which in turn determines the ice properties. *The most important conclusion to be drawn is that although the variation of ice temperature across the rime ice layer only modestly deviates from a linear profile, the same is not true of the formation temperature. As a result, ice property variation across the rime ice layer can be highly nonlinear*. The effect of this ice property variation on depth-dependent ice temperature is lower.

## 4. Conclusions

This study presents a rigorous formulation of the ice accumulation process in the rime ice phase, critically assessing the key assumptions of the existing icing models. Our analysis confirms that the widely used Messinger/Myers model, despite its simplifying assumptions, such as steady heat conduction, constant ice properties and neglect of sensible heat change of the ice layer over time, performs well in predicting both short-and long-term ice surface temperature evolution. At intermediate times, under conditions when rime ice transitions to glaze ice, the Myers model can result in an overprediction of ice surface temperature and correspondingly result in about 20% error in the estimation of critical time when rime ice transitions to glaze ice. Using the short- and long-time asymptotic analysis, we propose the MM model that is quite accurate in predicting ice thickness and ice surface temperature over a wide range of conditions.

We begin by formulating the unsteady one-dimensional heat conduction problem, initially assuming constant ice properties (density and thermal conductivity) while neglecting sublimation. Heat loss mechanisms considered include convective heat transfer and sensible heat loss to the impinging subcooled droplets, while heat gain mechanisms account for kinetic energy transfer from impinging droplets, latent heat release during freezing and aerodynamic heating. To properly account for all aspects of time dependence during ice layer growth, we account for the sensible heat change within the ice layer during its accretion (figure 3), an aspect that has been overlooked in the Messinger/Myers models. However, we find that including this term alone does not improve the Myers solution but actually yields less accurate estimates of ice surface temperature and thickness. Interestingly, the Myers model accurately captures both the initial rise and asymptotic final value of the ice surface temperature. This fortunate behaviour is due to the simultaneous neglect of both sensible heat as well as the other unsteady effects, along with the assumption of a linear temperature profile within the ice layer. As a result, in the constant property limit, the Myers model only slightly underpredicts surface temperature at intermediate times. Consequently, it marginally overestimates the transition time from rime to glaze ice and the corresponding rime ice thickness at this critical point.

Next, we extend our analysis by introducing variable ice properties. The density and thermal conductivity of ice are determined assuming (i) the ice properties at the time of formation depend linearly on the formation temperature (i.e. on the ice surface temperature at which the impinging droplets freeze to form the rime ice) and (ii) ice properties remain the same once formed, even as ice continues to accumulate on top. It is shown that the above-mentioned two processes do not lead to a linear variation in ice properties within the ice layer. We also incorporate the effect of sublimation, which requires modifying the mass balance since sublimation reduces ice thickness. The effects of sublimation and time-dependent ice surface density can be readily applied to the Myers model. However, the effect of variable thermal conductivity must be included by carefully considering its effect on increased heat loss.

Our analysis has identified three important non-dimensional parameters that control the regime of rime ice formation. The non-dimensional coefficient A measures the rate of total heat loss from the ice surface due to convective cooling, droplet heating, radiative cooling and sublimation, relative to sensible heat contained in the impinging droplets. The non-dimensional coefficient B measures the rate of total heat addition from kinetic heating, latent heat release due to freezing of the droplets, aerodynamic heating and residual sensible cooling due to droplets, relative to sensible heat contained in the impinging droplets. The non-dimensional coefficient ${\hat{\chi }}_{s}$ parameterizes the rate of sublimation compared to the rate of mass addition by impingement. We establish the range of possible values of A, B and ${\hat{\chi }}_{s}$ for varying external conditions influencing ice accretion, such as rate of droplet mass impingement, droplet impact velocity, the ambient and airfoil temperatures relative to the melting point and free-stream air velocity.

The following three regimes of rime ice accretion are identified: (R1) in this regime, rime ice does not transition to glaze ice, since the ice surface temperature reaches a steady value lower than the melting point. Also, mass loss by sublimation balances mass addition by droplet impaction, and the ice thickness reaches a constant value. This regime occurs when mass balance is achieved first as given by the requirement $1/{\hat{\chi }}_{s}<\left\{1,B/A\right\}$. (R2) In this regime also, rime ice does not transition to glaze ice, since the ice surface temperature reaches a steady value lower than the melting point. However, mass balance is not achieved, and rime ice continues to grow indefinitely at a constant rate. This regime occurs when the thermal balance is achieved first at the ice surface as given by the requirement $B/A<\left\{1,1/{\hat{\chi }}_{s}\right\}$. (R3) In this regime, rime ice transitions to glaze ice at a finite time. This regime occurs when ice surface temperature reaches the melting point before achieving mass or thermal balance as given by the requirement $1<\left\{B/A,1/{\hat{\chi }}_{s}\right\}$.

We introduce the MM model to accurately account for the effects of variable properties and sublimation. We show that the modified model perfectly recovers the exact numerical solution under all three regimes of ice accretion. Our findings indicate that when sublimation is small, particularly at higher droplet impingement rates, variable ice properties can have an appreciable influence in regime R3, and the Myers model can result in an overprediction of ice surface temperature. At lower impingement rates where sublimation becomes significant, the Myers model slightly overpredicts (or underpredicts) the ice surface temperature when heat loss at the ice surface is substantially smaller (or larger) than the heat addition mechanisms. However, we note that the overprediction or underprediction is generally small, and Myers’ model demonstrates good predictive accuracy. The proposed MM model is needed if one desires higher accuracy to match the higher fidelity of the flow simulation.

Although our analysis of ice accretion has been rigorous, some limitations of the present study must be stressed. We have assumed the droplet impingement rate to be constant, which may not always reflect conditions encountered in reality. Our analysis has also neglected a rigorous treatment of aerodynamic heating, particularly when the air layer near the ice surface acquires a temperature higher than the ambient. This can affect both convective heat transfer and the thermal state of small impacting droplets. It is also noted that the present work focuses exclusively on surface-level accretion and does not incorporate droplet-scale phenomena such as breakup, splashing or secondary impingement. While the effects of extreme icing are partially addressed through variations in mass impingement rate and thermal parameters, mixed-phase and transitional glaze icing regimes—particularly those involving water film dynamics—remain outside the scope of this work and will be addressed in future extensions of the work.

## Data Availability

This article has no additional data.

## Footnotes

^1^
The consistent values are given by $a_{1}=1/{\hat{h}}_{1}$ and $a_{2}=B({\hat{\chi }}_{s}{\hat{\Delta }}_{\rho }{\hat{h}}_{1})/(2{\hat{h}}_{1}^{2})$. Also, by setting ζ=1 in (3.15), we observe that $\theta_{I1}=B{\hat{h}}_{1}$ and $\theta_{I2}=-(AB{\hat{h}}_{1}^{2}+(1/2)B^{2}{\hat{h}}_{1}({\hat{\chi }}_{s}+{\hat{\Delta }}_{\rho }{\hat{h}}_{1})+(1/2){\hat{\Delta }}_{k}B^{2}{\hat{h}}_{1}^{2}.$

## Appendix A. Energy terms and their formulations

As stated in (2.2), the total heat loss from the ice layer surface, $A^{\prime }(T_{I}-T_{a})$, can be expressed as the sum of convective heat transfer ($Q_{c}$), sensible heat transfer to the impacting droplets ($Q_{d}$), sublimation heat loss ($Q_{s}$) and radiative heat loss ($Q_{r}$). The convective heat transfer represents the energy removed by airflow, which can be expressed as

(A 1)
$$\begin{array}{lcr} & Q_{c}=\underset{=q_{c}}{\underbrace{h_{c}}}(T_{I}-T_{a})\;, & \end{array}$$

where $h_{c}$ is the convective heat transfer coefficient. Sensible heat loss accounts for the thermal energy required to bring the impinging droplet to ice surface temperature [18,19]

(A 2)
$$\begin{array}{lcr} & \begin{array}{rl} Q_{d} & ={\dot{m}}_{im}\left(C_{p,w}(T_{f}-T_{a})+C_{p,i}(T_{I}-T_{f})\right)\\ & ={\dot{m}}_{im}(C_{p,w}-C_{p,i})(T_{f}-T_{a})+\underset{=q_{d}}{\underbrace{{\dot{m}}_{im}C_{p,i}}}(T_{I}-T_{a})\;. \end{array} & \end{array}$$

The first term on the right-hand side is a constant, and it is negative since the ambient temperature is below $T_{f}$. This term can be combined with the latent heat addition $Q_{L}$. The negative contribution of this sensible heat is generally much smaller than the latent heat. Sublimation leads to mass removal from the ice surface, and the associated heat loss can be expressed as [11,24]

(A 3)
$$\begin{array}{lcr} & Q_{s}=\underset{=q_{s}}{\underbrace{\frac{6.22\;h_{c}\;L_{s}e_{0}}{C_{p,a}\;p_{0}\;Le^{2/3}}}}(T_{I}-T_{a})\;, & \end{array}$$

where $e_{0}=27.3$ is the vapour pressure constant, $C_{P,a}$ is the specific heat of air, $p_{0}$ is ambient pressure, Le is Lewis number and $L_{s}$ is the latent heat of sublimation. The radiative heat loss follows Stefan–Boltzmann’s law, $\sigma \varepsilon_{i}(T_{I}^{4}-T_{a}^{4})$, where σ is Stefan–Boltzmann constant and $\varepsilon_{i}$ is the emissivity of ice. In the limit $(T_{I}-T_{a})\ll T_{a}$, we can express this as

(A 4)
$$\begin{array}{lcr} & Q_{r}=\sigma \varepsilon_{i}(T_{I}^{4}-T_{a}^{4})\approx \underset{=q_{r}}{\underbrace{4\sigma \;\varepsilon_{i}\;T_{a}^{3}}}(T_{I}-T_{a})\;. & \end{array}$$

Together, these terms define the total heat loss coefficient, $A^{\prime }=q_{c}+q_{d}+q_{s}+q_{r}$ from the ice layer.

The total heat addition to the ice layer, $B^{\prime }$, occurs through kinetic energy transfer ($Q_{k}$), latent heat release ($Q_{L}$) and aerodynamic heating ($Q_{a}$). The kinetic energy term accounts for the conversion of droplet kinetic energy into thermal energy upon collision and is given by

(A 5)
$$\begin{array}{lcr} & Q_{k}=\frac{1}{2}{\dot{m}}_{im}v_{im}^{2}\;, & \end{array}$$

where $v_{im}$ is the droplet impact velocity [7]. The latent heat term represents the energy released when liquid water transitions to solid ice and is given by

(A 6)
$$\begin{array}{lcr} & Q_{L}={\dot{m}}_{im}L_{f}\;, & \end{array}$$

where $L_{f}$ is the latent heat of fusion. Aerodynamic heating arises from the deceleration of high-speed airflow near the ice surface. This is usually given as [11,14]

(A 7)
$$\begin{array}{lcr} & Q_{a}=r\;q_{c}\;\frac{U_{\infty }^{2}}{2\;C_{p,a}}\;, & \end{array}$$

where $U_{\infty }$ is the free-stream velocity, and r is the adiabatic recovery factor, defined as $r=Pr^{1/2}$ for laminar flow and $r=Pr^{1/3}$ for turbulent flow, where Pr is the Prandtl number of air. These terms collectively define the total heat addition to the ice layer, $B^{\prime }=Q_{k}+Q_{L}+Q_{a}$, as defined in (2.2). In fact, it is more appropriate to combine aerodynamic heating with convective heating, since the effect of aerodynamic heating is to increase the air temperature near the ice surface above the ambient temperature.
